# *Pratylenchus brachyurus* is the prevalent root-lesion nematode in brazilian soybean fields, occurring singly and in mixed populations with *P*. *zeae, P. penetrans*, and *P*. *brasiliensis*

**DOI:** 10.3389/fpls.2026.1951549

**Published:** 2026-10-08

**Authors:** Francisco de Assis dos Santos Diniz, Eduardo S. G. Mizubuti, Pedro Henrique Lima Gomes, João Igor Araujo Valadares, Thaís Ribeiro Santiago

**Affiliations:** 1 Universidade Federal de Viçosa, Departamento de Fitopatologia, Viçosa, MG, Brazil; 2 Universidade de Brasília, Departamento de Fitopatologia, Brasília, DF, Brazil

**Keywords:** *Glycine max*, mixed infestations, *Pratylenchus zeae*, *Pratylenchus brachyurus*, *Pratylenchus brasiliensis*, resistance, root-lesion nematode

## Abstract

Root-lesion nematodes of the genus *Pratylenchus* are among the most economically important plant parasitic nematodes of soybean worldwide, yet their species diversity, distribution, and the genetic structure of the populations remain poorly resolved in Brazil. To address this knowledge gap, this study aimed to characterize the diversity, distribution, and genetic variation of *Pratylenchus* spp. associated with soybean in Brazil and evaluate soybean cultivar responses to the prevalent species. The study combined extensive field sampling with phylogenetic analyses based on independent molecular markers, morphological and morphometric characterization, and host-response assays. Soybean roots and rhizosphere soil were collected from 309 commercial fields across 16 major soybean-producing states. *Pratylenchus* spp. were detected in 95.8% (296/309) of the fields, from which 367 pure populations were established. This higher number reflects the separation of distinct morphotypes/species recovered from the same field samples. Sequencing of the D2–D3 expansion segments of 28S rDNA, Internal Transcribed Spacer rDNA, and partial mitochondrial Cytochrome c Oxidase subunit I revealed four species associated with soybean: *P. brachyurus* (detected in 292 fields), *P. zeae* (73 fields), *P. brasiliensis* (six fields), and *P. penetrans* (one field). This is the first report of *P. penetrans* associated with soybean in Brazil. Mixed-species infestations were detected in 72 fields, predominantly involving *P. brachyurus* and *P. zeae*. Three-species co-occurrence (*P. brachyurus*, *P. zeae*, and *P. penetrans*) was detected in one field. There was substantial intraspecific phylogenetic variation in *P. brachyurus* and *P. zeae*. Morphological and morphometric analyses largely corroborated molecular identification but highlighted persistent diagnostic overlap between *P. penetrans* and *P. brasiliensis*. Reproduction factors were highest for *P. brasiliensis* (Rf = 4.66–10.84) and *P. brachyurus* (Rf = 3.33–11.44), whereas *P. zeae* showed low Rf values (0.06–0.27). All soybean cultivars were susceptible to *P. brachyurus* and *P. brasiliensis* (Rf > 1.0) with the highest values estimated for ‘TMG 2833’ and ‘BMX Domínio’. *Pratylenchus* populations associated with soybean in Brazil are more taxonomically and genetically diverse than previously documented and the absence of effective resistance among current cultivars underscores the need for integrative diagnostics and region-specific management strategies that consider mixed infestations and genetically structured populations.

## 1 Introduction

Disruption of root function due to extensive necrosis is a hallmark of infection by species of *Pratylenchus*, a migratory endoparasitic nematode, that affects many plants, including soybean (*Glycine max* (L.) Merr.) (Thompson et al., 2008). The genus *Pratylenchus* Filipjev, 1936 forms a diverse and economically important group of plant parasitic nematodes that generally have a broad host range, worldwide distribution, and are capable of thriving across temperate, subtropical, and tropical agroecosystems. These characteristics underscore the importance of *Pratylenchus* spp. as major plant pests, despite the absence or rarity of sexual reproduction in some species (Castillo and Vovlas, 2007; Thiruchchelvan et al., 2025). For over a century this ecological success, combined with marked morphological variability, has challenged both basic biological studies such as species delimitation as well as disease management strategies, mainly those based on genetic resistance.

The genus *Pratylenchus* includes more than 100 described species (Gafur, 2020), yet identification remains difficult due to a limited set of morphological and morphometric diagnostic characters and the extensive overlap among closely related taxa. As a result, polyphasic identification frameworks integrating morphology, morphometrics, and molecular markers have become essential tools (Jones and Fosu-Nyarko, 2014). Sequencing of partial ribosomal (Internal Transcribed Spacer - ITS, D2–D3 of 28S, partial 18S) and mitochondrial (Cytochrome c Oxidase subunit I - COI) regions have markedly improved diagnostic accuracy and supported taxonomic revisions that clarified or redefined several species boundaries (Subbotin et al., 2008; De Luca et al., 2011, 2012; Jones and Fosu-Nyarko, 2014).

Despite these advances, molecular data remain unavailable for many nominal species, and the taxonomic status of several morphospecies is still unresolved, reflecting an urgent need for robust species-recognition frameworks (De Luca et al., 2010; Inserra et al., 2007; Janssen et al., 2017). In this context, phylogenetic analyses based on multiple molecular markers can contribute to species identification by providing complementary evidence from independent genomic regions, thereby supporting the recognition of cryptic or closely related species when combined with morphological and morphometric data (Janssen et al., 2017).

In Brazil, the impact of *Pratylenchus* spp. on soybean production is particularly significant, as the crop is cultivated on more than 45 million hectares (Embrapa, 2024). These nematodes are estimated to occur in up to 90% of soybean fields and may cause yield losses of up to 21%, representing a major agronomic and economic constraint (Lima et al., 2015). Although *P. brachyurus* (Godfrey, 1929) is the most prevalent species in soybeans, *P. zeae* Graham, 1951 is frequent in soybean-corn (*Zea mays* L.) – rotation areas (Goulart et al., 2003). Recent detection of a native species, *P. brasiliensis* Diniz et al., 2026 (Diniz et al., 2026), further suggests that agricultural expansion into distinct biomes may facilitate the adaptation and emergence of previously unrecognized species (Diniz et al., 2026). Despite their widespread importance, the soybean–*Pratylenchus* interactions remain insufficiently explored, and to date, no commercial soybean cultivars with confirmed resistance to these nematodes have been identified (Gouveia et al., 2025).

Morphological and morphometric traits remain central to species delimitation; however, overlapping characters among species, such as stylet morphology, vulva position, and the presence or absence of males, complicate diagnosis and often require advanced microscopy resources for reliable discrimination (Tarte and Mai, 1976; Thiruchchelvan et al., 2025). For example, the similarity between *Pratylenchus penetrans* (Cobb, 1917; Filipjev & Schuurmans Stekhoven, 1941) and *P. brasiliensis* illustrates how limited diagnostic traits and a shortage of trained taxonomists hinder accurate identification nationwide. Additional species such as *P. bolivianus* Corbett, 1983 have also been tentatively reported in soybean, indicating a richer species diversity than previously recognized (Ribeiro et al., 2013). This expanding diversity raises concerns about the emergence of new disease scenarios with substantial agronomic impact, especially given the polyphagous nature of the genus.

Despite growing evidence that soybean fields frequently harbor multiple *Pratylenchus* species and mixed populations, comprehensive large-scale surveys integrating morphological, molecular diagnostics, and host-nematode interaction remain scarce worldwide. In major soybean-producing regions, species diversity within this genus has been investigated primarily through regional surveys. In North America, species such as *P. penetrans*, *P. neglectus*, (Rensch 1924), *P. scribneri* Steiner, 1943, and *P. dakotaensis* Handoo et al., 2021 have been reported in soybean-based production systems (Niblack, 1992; Lewis et al., 1993; Ozbayrak et al., 2019; Chowdhury et al., 2022; Saikai and MacGuidwin, 2022). In Europe, *P. crenatus* Loof, 1960, *P. neglectus*, and *P. penetrans* have been reported from soybean fields, whereas in Asia, *P. coffeae* (Zimmermann, 1898) has been associated with soybean root rot under commercial conditions (Elhady et al., 2019; Wang et al., 2021; Li et al., 2022). These regional differences highlight the diversity of *Pratylenchus* assemblages associated with soybean. In Brazil, however, available studies remain limited in geographic coverage and diagnostic resolution, and a comprehensive national-scale assessment of species diversity and distribution is still lacking.

Early regional surveys based solely on morphological identification reported *P. brachyurus* in 82% of soybean samples collected in Tocantins (TO), northern Brazil, from a total of 11 samples (Lima et al., 2015). Subsequent surveys in central Brazil, including 12 isolates from Mato Grosso (MT) and Goiás (GO), confirmed the prevalence of *P. brachyurus* using ITS-5.8S rDNA sequences and BLAST comparisons with GenBank (Ribeiro et al., 2013). Nevertheless, these studies were geographically restricted and relied on a single diagnostic tool. Although host-response experiments have evaluated the interaction between soybean cultivars and *P. brachyurus* or *P. zeae*, such studies were not designed to assess species diversity or their distribution at regional or national scales (Silva et al., 2020). Large-scale, integrative surveys are essential to clarify species boundaries, resolve taxonomic inconsistencies, and characterize the diversity and distribution of *Pratylenchus* associated with soybean in Brazil. Therefore, this study aims to: (i) identify the species of *Pratylenchus* associated with soybean in 16 major soybean-producing states using morphological, morphometric, and phylogenetic analyses based on multiple molecular markers; (ii) assess their prevalence, geographic distribution, frequency of mixed-species populations, and intraspecific genetic variability; and (iii) evaluate soybean–*Pratylenchus* interactions and soybean susceptibility.

## 2 Materials and methods

### 2.1 Field sampling and nematode extraction

Between January 2021 and January 2023, samplings were conducted in all five macro-geographic regions of Brazil in which 16 major soybean-producing states are located. Fields were selected randomly, without prior information on nematode infestation and irrespective of the presence or absence of symptoms potentially associated with nematode infection. A total of 309 soybean root and soil samples were collected from: North region [Acre (AC), two samples; Pará (PA), two; and TO, two]; Northeast region [Bahia (BA), 29; Maranhão (MA), six; and Piauí (PI), one]; Central-West region [Federal District (DF), eight; GO, 56; MT, 28; and Mato Grosso do Sul (MS), 32]; Southeast region [Minas Gerais (MG), 36; São Paulo (SP), 27; and Espírito Santo (ES), five]; and South region [Paraná (PR), 40; Santa Catarina (SC), eight; and Rio Grande do Sul (RS), 27]. These samples are part of a previous survey conducted by Diniz et al. (2026).In that earlier study, *P. brasiliensis* was first reported from four Brazilian municipalities: Primavera do Leste (MT), Itapiratins (TO), Luziânia (GO), and Brasiléia (AC). Because these four samples had already been analyzed and published by Diniz et al. (2026), they were not considered new samples in the present study. Their previously published data were used only for comparative purposes, where appropriate.

In each field, four random subsamples of 300 g of soil and soybean roots were collected at a depth of 20–30 cm, from sampling points located approximately 5 m apart within the field and pooled into a single composite sample. All samples were obtained from georeferenced points, placed in insulated cooler boxes, and transported to the Epidemiology Laboratory at the University of Brasília (UnB), Federal District, Brazil.

To confirm the presence of *Pratylenchus* spp., field-collected infested root fragments and associated soil were transferred to pots containing sterilized substrate, into which soybean seedlings cv. ‘BMX Domínio’ were subsequently planted and maintained under greenhouse conditions for two months at 25 ± 2 °C. At the end of the incubation period, nematodes were extracted from soybean roots following the method of Coolen and D'Herde (1972).

### 2.2 Purification and characterization of populations

After nematodes were extracted from soybean roots, specimens of *Pratylenchus* were visually sorted into groups with the aid of a stereoscope (Leica EZ4; 8X–35X magnification) and an optical microscope (Zeiss Primo Star; 40X objective). Morphotypes were distinguished based on readily observable morphological differences, including stylet morphology, lip-region configuration, vulval position, tail shape, and the presence or absence of males. Individual nematodes representing each morphotype were inoculated separately onto carrot discs to establish pure populations. For each morphotype, five individuals were transferred individually to carrot discs per sample.

Pure populations were established using carrot disc cultures, as previously described (Diniz et al., 2026). Pure populations were consistently established from individual gravid females selected from each morphotype, irrespective of the presence or absence of males in the original sample. Carrot discs were incubated in a BOD chamber set at 25 ± 2 °C under continuous darkness for 80 days. The five individually inoculated cultures served as independent attempts to establish each morphotype. After successful multiplication and confirmation of morphological uniformity, one representative pure culture was retained for each morphotype within each field sample, whereas the remaining replicate cultures were not included in subsequent analyses. These pure populations were subsequently characterized using phylogenetic, morphological, and morphometric methods.

### 2.3 Phylogenetic characterization of pure *Pratylenchus* populations collected in soybean-producing macroregions of Brazil

Genomic DNA from all pure populations was extracted following the protocol of Diniz et al. (2026). Each PCR reaction was prepared in a final volume of 12.5 μl, containing 1 μl of genomic DNA (~20 ng), 6.25 μl of DreamTaq PCR Master Mix (2×) (Thermo Scientific Fermentas Ltd), 0.3 μl of each primer (10 μM), and 4.65 μL of nuclease-free water. The amplification employed three primer sets targeting distinct genomic regions: forward D2A (5′-ACAAGTACCGTGAGGGAAAGTTG-3′) and reverse D3B (5′-TCGGAAGGAACCAGCTACTA-3′) for the D2–D3 expansion segment of 28S rDNA (Nunn, 1992); forward Vrain2F (5′-CTTTGTACACACCGCCCGTCGCT-3′) and reverse Vrain2R (5′-TTTCACTCGCCGTTACTAAGGGAATC-3′) for the ITS rDNA (Vrain et al., 1992); and forward JB3 (5′-TTTTTTGGGCATCCTGAGGTTTAT-3′) and reverse JB4.5 (5′-TAAAGAAAGAACATAATGAAAATG-3′) for partial mtDNA COI (Derycke et al., 2010).

All PCR amplicons were examined on 1% agarose gels and subsequently sequenced at the Laboratory of Epidemiology and Control of Phytopathogens, UnB, using an Oxford Nanopore Technologies (ONT) PromethION platform equipped with an R10.4.1 flow cell. PCR products corresponding to the COI, ITS, and 28S regions obtained from each purified *Pratylenchus* population were individually indexed using the Rapid Barcoding Kit 96 (SQK-RBK114.96, chemistry v14; ONT), following the manufacturer’s protocol with minor modifications. Amplicons from each population were assigned unique barcodes, allowing multiple populations and loci to be multiplexed within a single sequencing library. The barcoded products were subsequently pooled, purified, and prepared for sequencing following the ONT library preparation protocol.

Sequencing was performed using the standard PromethION acquisition settings recommended for R10.4.1 chemistry. Raw electrical signals were converted into nucleotide sequences by basecalling with Dorado v0.8.0 (ONT), using the super-accuracy model optimized for R10.4.1 chemistry. Reads were subsequently demultiplexed according to their barcode sequences using the Dorado demux module. Reads for which a barcode could not be confidently assigned were excluded from downstream analyses, and adapter and barcode sequences were removed during demultiplexing and read processing.

Demultiplexed reads were quality filtered, retaining only sequences with a Phred quality score >10 (Q > 10). Reads were then assigned to the expected amplicon and screened for length and sequence quality to exclude incomplete or nonspecific products. For each population and molecular marker, a minimum depth of 1,000 quality-filtered reads was required for consensus-sequence reconstruction.

Consensus sequences for the COI, ITS, and 28S amplicons were generated independently in Geneious Prime v2024.0.7 from the corresponding quality-filtered reads, with the predominant nucleotide assigned at each position. Sites with insufficient support or ambiguous base calls were manually inspected against the underlying read alignments, and only high-quality consensus sequences with complete target-region coverage and no unresolved ambiguities were retained. The curated consensus sequences were then aligned with reference sequences from previous studies (Duncan et al., 1999; Subbotin et al., 2008; De Luca et al., 2010, 2011; Palomares-Rius et al., 2010; Taheri et al., 2013; Wang et al., 2015; Pili et al., 2016; Troccoli et al., 2016) using MUSCLE (Edgar, 2004).

To infer phylogenetic relationships, the best-fit evolutionary models were determined in jModelTest 0.1.1 (Posada, 2008) under the Akaike Information Criterion (AIC). Bayesian inference (BI) analyses were subsequently conducted in MrBayes 3.1.2 (Huelsenbeck and Ronquist, 2001). Each dataset was analyzed with four Markov chains run for 1,000,000 generations, sampling every 100 generations. Two independent runs were performed per marker, and after discarding the first 25% of trees as burn-in, consensus trees were generated.

### 2.4 Morphological and morphometric characterization of pure *Pratylenchus* populations from soybean-producing macroregions of Brazil

Morphological and morphometric characterization of pure populations of *Pratylenchus* was conducted on freshly extracted nematodes from 63 pure populations collected across the major soybean-producing regions of Brazil. These 63 populations were selected from the 367 pure populations available in the survey to provide broad geographic representation across the sampled states and to encompass the molecular diversity observed among populations based on the markers used in this study. Whenever possible, populations representing distinct geographic origins and different molecular profiles were prioritized to capture the overall phenotypic and genetic variability present in the dataset.

Observations and photomicrographs were obtained using a Leica DM 2500 optical microscope equipped with a DFC 490 digital camera. Particular attention was given to the shape and structure of the stylet and its basal knobs, the degree of esophageal overlap with the intestine, the configuration of the labial region, the presence and shape of the spermatheca, and the diversity of tail morphologies. The presence of males and the occurrence of a bursa were also recorded whenever observed.

Morphometric and morphological datasets were generated from measurements of 10 adult females per population, and 10 adult males were also measured when available. Morphometric ratios were calculated according to De Man (1880). The complete list of morphometric variables and their abbreviations is provided in **Table 1**.

Table 1Morphometric characteristics of *Pratylenchus brachyurus*, *P. zeae*, *P. brasiliensis* and *P. penetrans* populations collected from soybean-growing areas in Brazil.

**Table:** 

(A)								
Characters	*P. brachyurus* (51)	*P. brachyurus* Godfrey (1929)	*P. zeae* (09)	*P. zeae* Graham (1951)	*P. brasiliensis* (02)	*P. brasiliensis* Diniz et al. (2026)	*P. penetrans* (01)	*P. penetrans* Cobb (1917) Filipjev & Schuurmans Stekhoven, 1941
L	649.87 ± 52.62 (530.64 - 787.94)	560 (520 – 600)	511.22 ± 51.62 (434.56 - 607.36)	540 (360 – 580)	637.85 ± 50.05 (518.69 - 805.49)	622.29 ± 44.98 (562.74 - 671.98)	710.94 ± 57.09 (625.28 -779.17)	343-811
a	20.60 ± 1.57 (18.11 - 24.80)	20 (18 – 23)	18.94 ± 1.61 (17.31 - 21.46)	27 (25-30)	22.47 ± 1.94 (17.98 - 25.55)	24.55 ± 1.45 (23.09 - 26.42)	22.24 ± 2.26 (18.19 - 26.04)	
b’	4.25 ± 0,27 (3.75 - 4.91)	3.9 (3.4 -5.2)	4.48 ± 0.32 (4.05 - 5.09)	6.5 (5.4-8)	4.66 ± 0.33 (3.57 - 5.68)	5.03 ± 0.35 (4.69 - 5.48)	4.93 ± 0.33 (4.57 - 5.48)	
c	19.77 ± 1.13 (16.44 - 21.91)	19 (16 – 23)	17.83 ± 1.55 (15.83 - 21.16)	15.2 (17-21)	19.19 ± 1.45 (15.83 - 21.14)	20.12 ± 1.39 (18.22 - 21.32)	18.81 ± 0.79 (17.26 - 19.77)	15-24
c’	2.00 ± 0.21 (1.11 - 2.39)	–	2.00 ± 0.25 (1.64 - 2.48)	–	2.34 ± 0.17 (1.96 - 2.56)	2.26 ± 0.2 (2.1 - 2.56)	2 ± 0.17 (1.56 - 2.19)	
V	85.73 ± 1.06 (79.66 - 87.27)	86 (84 – 87)	71.62 ± 1.07 (70.36 – 73.30)	71 (68-76)	79.97 ± 1.03 (77.72 - 83.05)	79.28 ± 1.15 (77.76 - 80.22)	79.53 ± 1.21 (78.22 - 81.64)	75-84
HL	2.98 ± 0.19 (2.61 - 3.76)	–	2.95 ± 0.42 (2.52 - 3.96)	–	2.96 ± 0.13 (2.59 - 3.49)	3.44 ± 0.17 (3.25 - 3.64)	3.03 ± 0.23 (2.66 - 3.41)	
ØL	9.00 ± 0.31 (8.28 - 9.63)	–	7.81 ± 0.40 (7.06 - 8.15)	–	8.45 ± 0.43 (7.82 - 9.46)	8.62 ± 0.43 (8.3 - 9.25)	9 ± 0.56 (8.3 - 10.23)	
St	21.23 ± 0.66 (19.39 - 22.46)	19 (18 – 21)	16.64 ± 0.55 (15.35 - 17.21)	15.5 (15-17)	17.14 ± 0.38 (15.81 - 18.61)	17.45 ± 0.47 (16.84 - 17.97)	17.76 ± 0.74 (16.20 - 18.73)	15-17
ØbSt	5.44 ± 0.36 (4.49 - 6.76)	–	4.75 ± 0.41 (4.02 - 5.30)	–	4.14 ± 0.08 (3.23 - 4.83)	4.38 ± 0.31 (4.11 - 4.81)	4.67 ± 0.26 (4.43 - 5.22)	
AbSt	3.745± 0.25 (2.87 - 4.23)	–	2.60 ± 0.24 (2.06 - 2.88)	–	2.23 ± 0.06 (1.93 - 2.45)	2.49 ± 0.36 (2.08 - 2.95)	2.60± 0.32 (2.05 - 3.07)	
DEA	105.66 ± 7.18 (90.90- 128.05)	–	81.49 ± 8.85 (73.24 - 97.56)	–	98.62 ± 5.44 (89.66 - 113.20)	95.47 ± 6.24 (89.61 - 102.87)	102.98 ± 7.53 (- 91.35 - 111.6)	
CE	153.55 ± 7.25 (140.50 - 175.77)	–	114.21 ± 5.42 (101.43 - 119.71)	–	137.89 ± 11.82 (114.69 - 166.02)	125.14 ± 10.22 (115.9 - 139.65)	143.27 ± 13.66 (122.82 - 160.53)	
PGO	57.10 ± 4.33 (43.77 - 66.06)	–	30.31 ± 3.43 (25.90 - 36.85)	–	29.95 ± 4.97 (22.65 - 45.06)	41.16 ± 3.97 (35.79 - 45.29)	40.95 ± 6.86 (30.85 - 48.42)	
PUS	23.92 ± 2.75 (18.71 - 32.60)	–	22.95 ± 1.90 (20.71 - 26.22)	–	29.30 ± 5.16 (19.90 - 40.55)	29.58 ± 3.93 (24.89 - 34.09)	25.46 ± 5.98 (15.44 - 32.51)	
Øc	32.02 ± 3.02 (24.67 - 39.58)	–	27.44 ± 3.14 (21.12 - 31.59)	–	28.66 ± 4.62 (20.44 - 36.25)	25.84 ± 1.96 (22.95 - 27.34)	32.27 ± 4.13 (25.66 - 36.97)	
ØLVU	25.71 ± 1.84 (21.13 - 29.62)	–	24.67 ± 2.72 (19.89 - 28.72)	–	25.14 ± 1.23 (18.99 - 30.16)	22.69 ± 1.64 (20.37 - 24.17)	28.29 ± 2.38 (24.36 - 31.37)	
Øa	16.40 ± 0.88 (14.35 - 17.88)	–	14.79 ± 1.82 (11.47 - 17.40)	–	14.91 ± 1.97 (13.22 - 17.39)	13.58 ± 1.26 (12.11 - 15.14)	18.94 ± 1.42 (17.12 - 21.82)	
CT	32.87 ± 2.45 (28.49 - 37.85)	–	29.22 ± 4.49 (24.60 - 38.91)	–	34.73 ± 1.99 (31.25 - 38.11)	30.63 ± 3.09 (26.61 - 33.98)	37.82 ± 3.06 (34.01 - 42.68)	
HC	5.11 ± 0.66 (3.18 - 6.19)	–	0 ± 0 (0 - 0.00)	–	0 ± 0.00 (0.00 - 0.00)	0 ± 0.00 (0.00 - 0.00)	0 ± 0,00 (0,00 - 0,00)

**Table:** 

(B)				
Characters	*P. brachyurus* (02)	*P. brasiliensis* Diniz et al. (2026)	*P. brasiliensis* (02)	*P. penetrans* (01)
L	519.82 ± 46.25 (458.92 - 594.93)	518.12 ± 18.16 (497.84 - 540.61)	504.12 ± 20.48 (474.39 - 538.32)	635.68 ± 16.68 (613.70 - 663.17)
a	23.75 ± 1.67 (20.83 - 26.05)	25.75 ± 1.58 (24.23 - 27.26)	25.33 ± 2.28 (23.06 - 28.88)	27.19 ± 0.95 (25.84 - 28.18)
b’	3.90 ± 0.40 (3.33 - 4.42)	4.35 ± 0.30 (3.97 - 4.68)	4.11 ± 0.33 (3.58 - 4.49)	4.90 ± 0.27 (4.59 - 5.32)
c	20.41± 3.78 (16.77- 24.39)	20.32 ± 0.46 (19.82 - 20.88)	19.01 ± 0.76 (18.10 - 20.54)	19.60 ± 0.69 (18.71 - 20.54)
c’	1.99 ± 0.49 (1.46 - 2.47)	2.04 ± 0.26 (1.75 - 2.35)	2.23 ± 0.18 (1.92 - 2.50)	2.31 ± 0.11 (2.18 - 2.48)
HL	2.70 ± 0.45 (2.24 - 3.55)	3.15 ± 0.26 (2.79 - 3.38)	2.72 ± 0.30 (2.27 - 3.26)	2.89 ± 0.33 (2.29 - 3.22)
ØL	7.74 ± 0.37 (6.95 - 8.21)	8.02 ± 0.28 (7.65 - 8.32)	7.38 ± 0.59 (6.63 - 8.20)	8.18 ± 0.43 (7.57 - 8.85)
St	18.98 ± 1.17 (16.94 - 20.91)	16.61 ± 0.54 (15.84 - 17.01)	16.25 ± 0.40 (15.52 - 16.83)	16.06 ± 1.03 (14.05 - 16.96)
ØbSt	3.58 ± 0.67 (2.82 - 4.61)	3.47 ± 0.21 (3.25 - 3.74)	3.79 ± 0.42 (3.19 - 4.25)	3.50 ± 0.48 (2.93 - 4.27)
AbSt	3.07 ± 0.50 (2.42 - 4.11)	2.18 ± 0.30 (1.78 - 2.52)	2.12 ± 0.24 (1.73 - 2.48)	2.2 ± 0.29 (1.70 - 2.48)
DEA	94.99 ± 6.96 (84.48 - 103.47)	88.39 ± 5.18 (80.81 - 91.88)	86.38 ± 4.45 (79.45 - 91.71)	98.55 ± 5.54 (89.55 - 106.47)
CE	132.8 ± 8.67 (118.00 - 146.90)	120.85 ± 11.86 (109.41 - 136.24)	123.25 ± 7.91 (114.05 - 135.78)	129.78 ± 8.38 (115.37 - 136.18)
PGO	34.33 ± 7.99 (28.10 - 45.99)	3.47 ± 8.90 (30.66 - 43.25)	28.22 ± 9.29 (20.54 - 44.04)	29.68 ± 5.32 (22.01 - 35.70)
Øc	26.63 ± 3.71 (23.56 - 31.07)	20.47 ± 1.76 (18.46 - 21.70)	19.99 ± 1.23 (17.61 - 21.57)	23.39 ± 0.83 (22.08 - 24.57)
Øa	13.5 ± 1.72 (11.45 - 16.14)	12.76 ± 1.36 (11.64 - 14.60)	11.92 ± 0.75 (11.11 - 13.45)	14.01 ± 0.25 (13.70 - 14.35)
CT	26.63 ± 3.71 (23.56 - 31.07)	25.53 ± 1.29 (24.52 - 27.36)	26.56 ± 1.40 (24.84 - 29.01)	32.46 ± 1.19 (30.89 - 33.93)
Lspic	18.46 ± 1.51 (16.90 - 20.65)	16.27 ± 0.83 (15.22 - 17.23)	18.16 ± 1.48 (15.53 - 19.95)	17.78 ± 1.65 (15.50 - 19.61)
Øspic	3.75 ± 0.79 (3.07 - 5.26)	3.50 ± 0.38 (2.95 - 3.79)	3.68 ± 0.57 (3.04 - 4.64)	2.68 ± 0.62 (1.61 - 3.48)
gub	–	7.96 ± 0.51 (7.60 - 8.32)	–	–

All measurements are given in µm. Data are presented as mean ± standard deviation (range) based on measurements of 10 individuals per population. The number of populations evaluated for each species is indicated in parentheses. (A) Females; (B) Males.

L, body length; V, vulval position relative to the anterior end, expressed as a percentage of total body length; a, body length to maximum body width ratio; b′, body length to distance from the anterior end to the base of the esophageal glands ratio; c, body length to tail length ratio; c′, tail length to anal body diameter ratio; HL, lip height; ØL, lip diameter; St, stylet length; ØbSt, stylet knob width; AbSt, stylet knob height; DEA, distance from the anterior end to the excretory pore; CE, total esophageal length; PGO, pharyngeal gland overlap; PUS, post-uterine sac length; Øc, maximum body width; ØLVU, vulval body diameter; Øa, body width at the anal region; CT, tail length; HC, hyaline tail length; Lspic, spicule length (along the arc); Øspic, spicule width; gub, gubernaculum length.

Comparative analyses were conducted among the different morphotypes identified. To assess morphometric variation among populations, principal component analysis (PCA) was performed using R software (version 4.4.1) with the packages FactoMineR and Factoextra. PCA was used to identify the most informative variables, determine the optimal number of morphometric clusters, and assign each population to its corresponding morphological group. Morphometric data for *P. penetrans* used in the PCA were obtained from isolates previously characterized by Janssen et al. (2017) and Gil et al. (2021), whereas data for *P. brasiliensis* comprised four populations reported by Diniz et al. (2026).

### 2.5 Multiplication of prevalent *Pratylenchus* species in commercial soybean cultivars

This experiment aimed to evaluate the multiplication rate of prevalent *Pratylenchus* species in eight major commercial soybean cultivars cultivated in Brazil: ‘BMX Desafio’, ‘BMX Domínio’, ‘BMX Foco’, ‘BMX Guepardo’, ‘BMX Olimpo’, ‘CZ37B43’, ‘Monsoy 8349’, and ‘TMG 2833’. Two independent experiments were conducted under greenhouse conditions using the same experimental design and procedures. Each experiment followed a completely randomized design in an 8 × 3 factorial arrangement (cultivars × nematode species), totaling 24 treatments, with eight replicates per treatment. The three isolates used for soybean plant inoculation were UnBPratySoy09, UnBPratySoy29, and UnBPratySoy113, which were identified in this study as *P. brasiliensis*, *P. zeae*, and *P. brachyurus*, respectively.

Soybean seeds were sown in 1.5-L plastic pots containing a sterilized mixture of soil, sand, and substrate (Bioplant^®^, 1:1:1, v/v/v), previously autoclaved at 121 °C for 2 h in two days. Plants were inoculated at the V1 growth stage with an initial population (Pi) of 700 mixed-stage nematodes per plant. The plants were maintained for 75 days under greenhouse conditions at the Institute of Biological Sciences, UnB, at 25 ± 2 °C.

Seventy-five days after inoculation, nematodes were extracted from the root systems using the method described by Coolen and D'Herde (1972). The final nematode population (Pf) was quantified, and the reproduction factor (RF = Pf/Pi) was calculated. An Rf value greater than 1 indicates susceptibility of cultivars.

Rf data were subjected to analysis of variance (ANOVA). When significant effects were detected, means were compared using Fisher’s least significant difference (LSD) test at the 5% probability level. The effects of soybean cultivar, *Pratylenchus* species, and their interaction were evaluated in accordance with the factorial experimental design.

## 3 Results

Of the 309 soybean root samples collected across 16 soybean-producing states in Brazil (Diniz et al., 2026), *Pratylenchus* specimens were successfully extracted and identified in 296 samples (95.8%). In the remaining 13 samples (4.2%), no individuals of the genus were recovered after nematode extraction, indicating their absence or extremely low population levels at the time of sampling.

The 296 positive samples were widely distributed confirming the broad geographic range of *Pratylenchus* in Brazil. *Pratylenchus* spp. was found in soybean fields from all surveyed states. In the North region, two samples were positive for *Pratylenchus* in AC, one in PA, and two in TO. In the other regions the frequency was also high: Northeast (BA, 29 samples; MA, four; PI, one), Central-West (DF, eight; GO, 54; MT, 28; MS, 32), Southeast (MG, 34; SP, 26; ES, five), and South (PR, 36; SC, eight; RS, 26). Samples that were positive for *Pratylenchus* were coded UnBPratySoy01 through UnBPratySoy296, reflecting the full set analyzed in this study (**Figure 1**; **Supplementary Table S1**).

**Figure 1:**
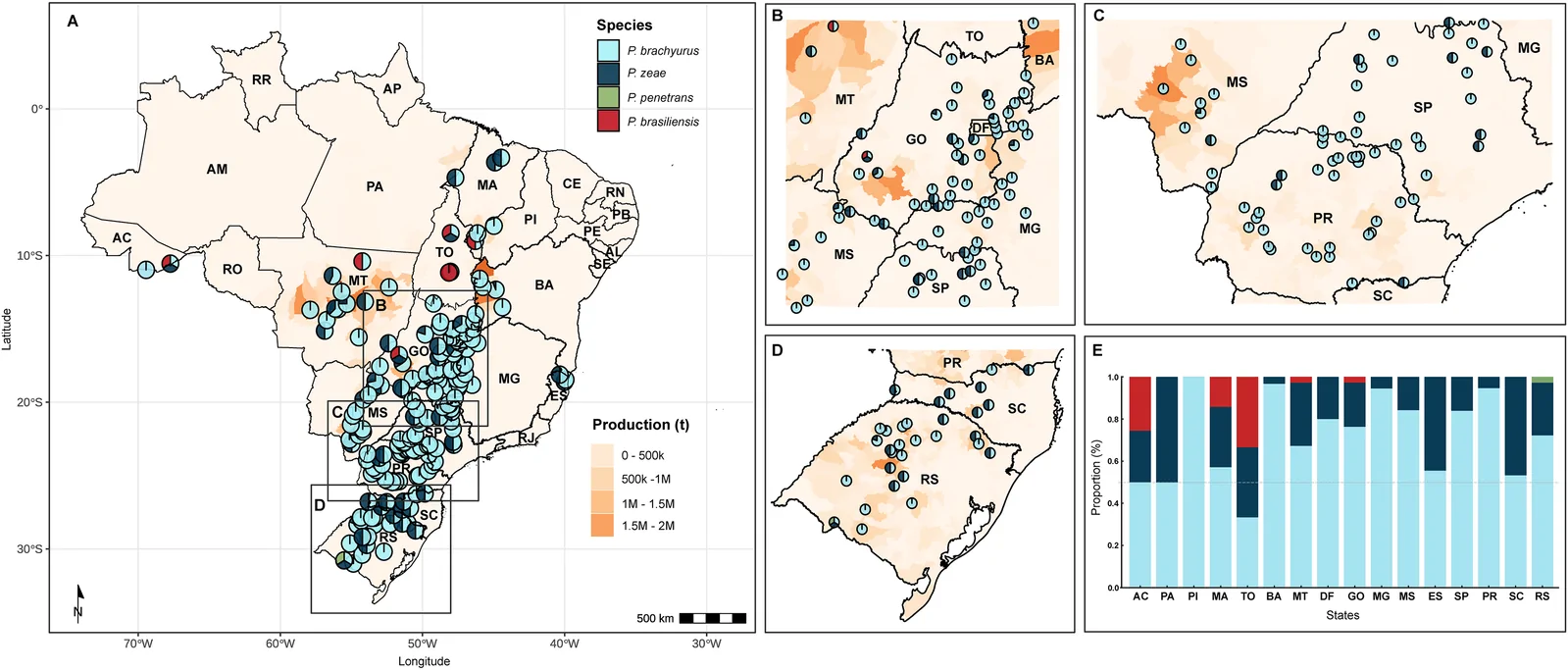
General overview of the geographic distribution of *Pratylenchus* populations sampled from soybean-producing areas in Brazil. **(A–D)** Sampling sites (circles) represent municipalities across five macro-regions and 16 states: North (Acre [AC], 2 samples; Pará [PA], 1; Tocantins [TO], 2), Northeast (Bahia [BA], 29; Maranhão [MA], 4; Piauí [PI], 1), Central-West (Federal District [DF], 8; Goiás [GO], 54; Mato Grosso [MT], 28; Mato Grosso do Sul [MS], 32), Southeast (Minas Gerais [MG], 34; São Paulo [SP], 26; Espírito Santo [ES], 5), and South (Paraná [PR], 36; Santa Catarina [SC], 8; Rio Grande do Sul [RS], 26) and data from Diniz et al. (2026), which include four additional samples, one collected in each of the following states: MT, TO, GO, and AC. Panels **(B–D)** are zoomed out views for the areas delimited by the squares in **(A)** Populations were collected between January 2021 and March 2023 and identified based on phylogenetic analyses of the D2–D3 expansion segments of the 28S rDNA gene, the internal transcribed spacer (ITS) region, and partial mitochondrial cytochrome c oxidase subunit I (COI) sequences. Background shading indicates soybean productivity levels, with darker orange tones corresponding to higher yields. Productivity data were obtained from the Brazilian Institute of Geography and Statistics (IBGE). **(E)** Barplot of the geographic distribution of *Pratylenchus* species across 16 states in Brazil and data from Diniz et al. (2026).

From these 296 positive samples, a total of 367 pure *Pratylenchus* populations were established, as more than one morphotype was observed in several field samples. Following DNA extraction, PCR amplification, and sequencing of three reference loci (28S rDNA, ITS rDNA, and mtDNA COI), multilocus phylogenetic analyses incorporating sequences of 28S rDNA, ITS rDNA, and COI revealed clear and concordant species-level resolution. All sequences derived from the 367 populations formed well-supported clusters with validated GenBank reference sequences for *P. brachyurus*, *P. zeae*, *P. brasiliensis*, and *P. penetrans* (**Figures 2**–**4**; **Supplementary Table S1**).

**Figure 2:**
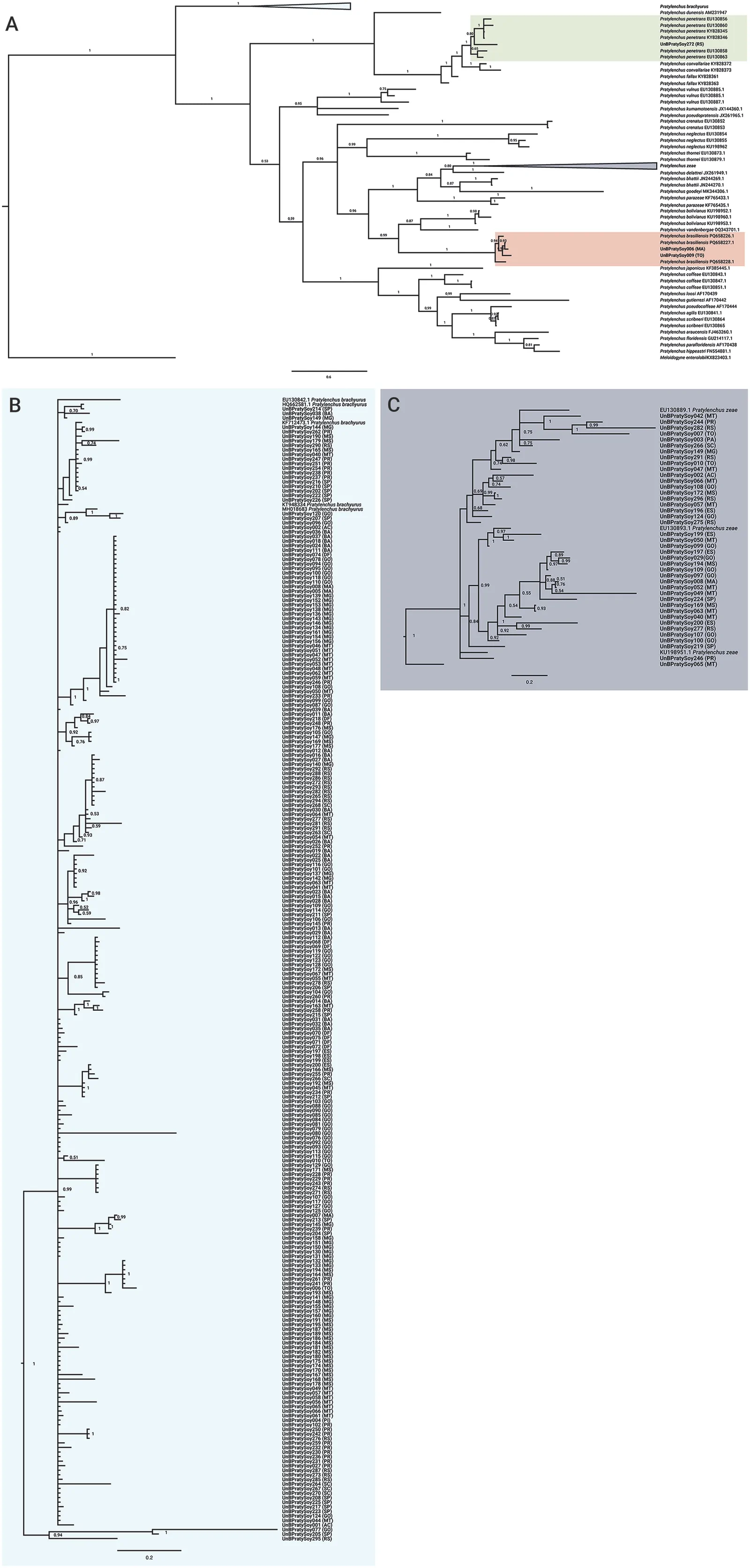
**(A)** Bayesian phylogenetic reconstruction inferred from sequences of the D2–D3 expansion segments of the 28S rDNA gene for *Pratylenchus* populations collected from soybean fields across Brazil. Samples were obtained from five macroregions encompassing 16 states: North (Acre [AC], Pará [PA], Tocantins [TO]), Northeast (Bahia [BA], Maranhão [MA], Piauí [PI]), Central-West (Federal District [DF], Goiás [GO], Mato Grosso [MT], Mato Grosso do Sul [MS]), Southeast (Minas Gerais [MG], São Paulo [SP], Espírito Santo [ES]), and South (Paraná [PR], Santa Catarina [SC], Rio Grande do Sul [RS]), between January 2021 and March 2023. Posterior probability values are shown at the corresponding nodes, and branch lengths are proportional to the number of substitutions per site, as indicated by the scale bar. The tree was rooted using *Meloidogyne enterolobii*. Sequences generated in the present study are shown in bold and labeled with their state abbreviations, whereas reference sequences were retrieved from GenBank. Enlarged views highlight the polytomy observed within the *Pratylenchus brachyurus* **(B)** and *P. zeae* **(C)** lineages.

**Figure 3:**
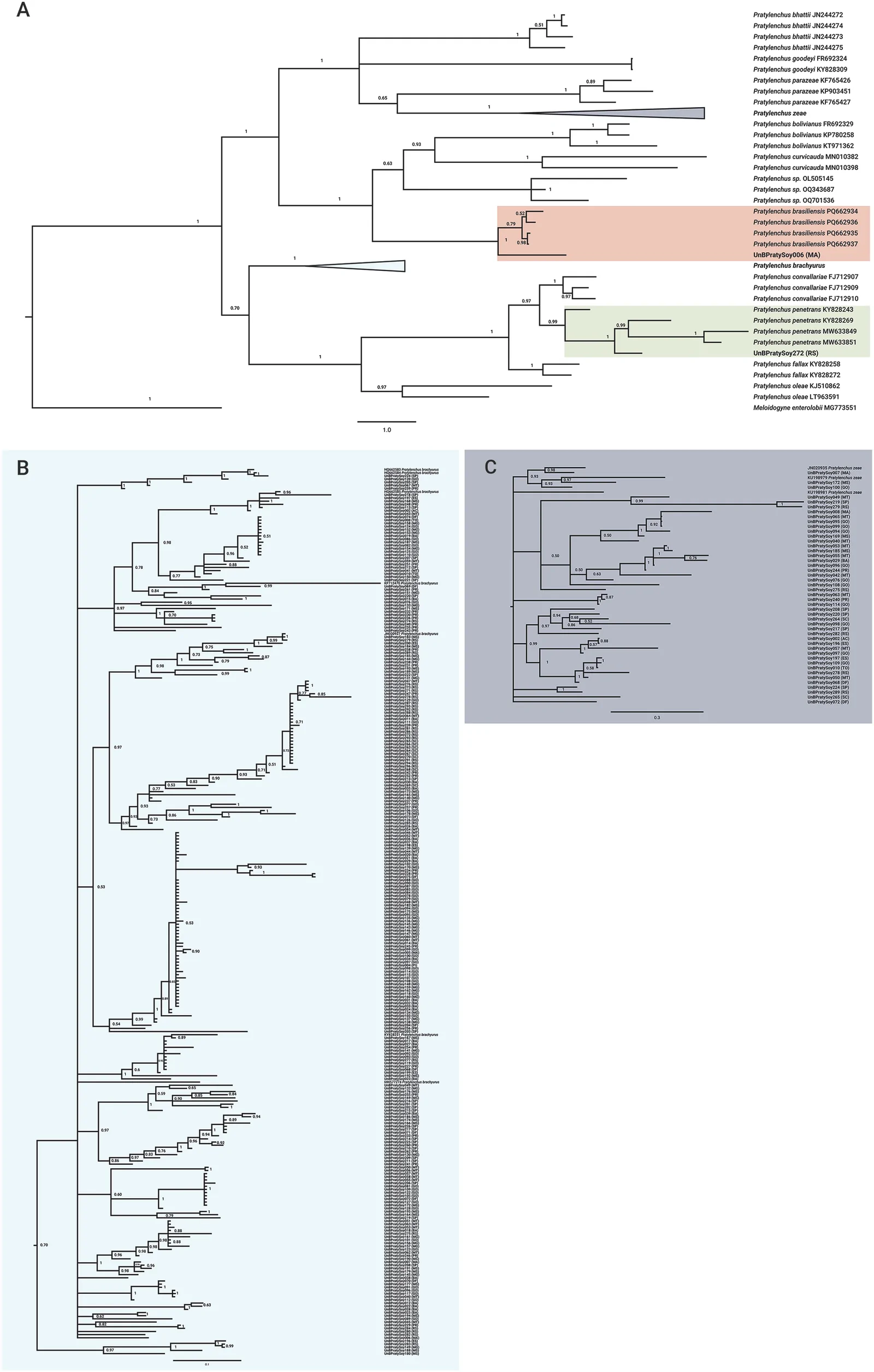
**(A)** Bayesian phylogenetic reconstruction inferred from sequences of the internal transcribed spacer (ITS) rDNA gene for *Pratylenchus* populations collected from soybean fields across Brazil. Samples were obtained from five macroregions encompassing 16 states: North (Acre [AC], Pará [PA], Tocantins [TO]), Northeast (Bahia [BA], Maranhão [MA], Piauí [PI]), Central-West (Federal District [DF], Goiás [GO], Mato Grosso [MT], Mato Grosso do Sul [MS]), Southeast (Minas Gerais [MG], São Paulo [SP], Espírito Santo [ES]), and South (Paraná [PR], Santa Catarina [SC], Rio Grande do Sul [RS]), between January 2021 and March 2023. Posterior probability values are shown at the corresponding nodes, and branch lengths are proportional to the number of substitutions per site, as indicated by the scale bar. The tree was rooted using *Meloidogyne enterolobii*. Sequences generated in the present study are shown in bold and labeled with their state abbreviations, whereas reference sequences were retrieved from GenBank. Enlarged views highlight the polytomy observed within the *Pratylenchus brachyurus* **(B)** and *P. zeae* **(C)** lineages.

**Figure 4:**
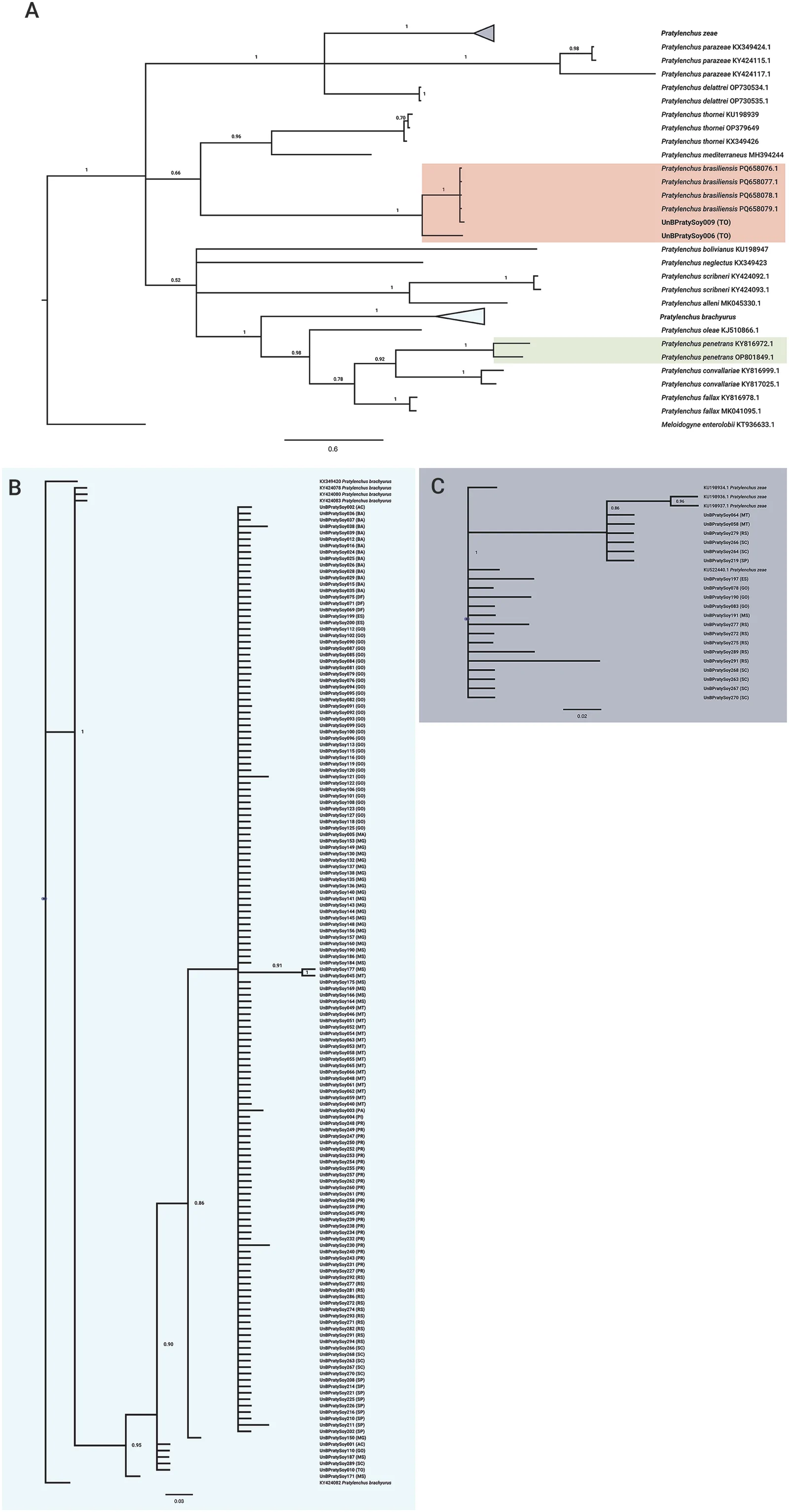
**(A)** Bayesian phylogenetic reconstruction inferred from sequences of the cytochrome oxidase I (COXI) mitochondrial gene for *Pratylenchus* populations collected from soybean fields across Brazil. Samples were obtained from five macroregions encompassing 16 states: North (Acre [AC], Pará [PA], Tocantins [TO]), Northeast (Bahia [BA], Maranhão [MA], Piauí [PI]), Central-West (Federal District [DF], Goiás [GO], Mato Grosso [MT], Mato Grosso do Sul [MS]), Southeast (Minas Gerais [MG], São Paulo [SP], Espírito Santo [ES]), and South (Paraná [PR], Santa Catarina [SC], Rio Grande do Sul [RS]), between January 2021 and March 2023. Posterior probability values are shown at the corresponding nodes, and branch lengths are proportional to the number of substitutions per site, as indicated by the scale bar. The tree was rooted using *Meloidogyne enterolobii*. Sequences generated in the present study are shown in bold and labeled with their state abbreviations, whereas reference sequences were retrieved from GenBank. **(B)** Enlarged views highlight the polytomy observed within the *Pratylenchus brachyurus* **(B)** and *P. zeae* **(C)** lineages.

### 3.1 General overview of the distribution and prevalence of *Pratylenchus* species across Brazilian states

*Pratylenchus brachyurus* was the prevalent species, occurring in 292 samples and detected in every surveyed state. *Pratylenchus brachyurus* was absent only from samples UnBPratySoy09, UnBPratySoy42, UnBPratySoy83, and UnBPratySoy244. *Pratylenchus zeae* was identified in 73 populations, while *P. brasiliensis* and *P. penetrans* were rare, occurring in only six populations: two samples in this study (UnBPratySoy06 and UnBPratySoy09) and four reported by Diniz et al. (2026); and one population (UnBPratySoy272), respectively (**Figures 1A–E**; **Supplementary Table S1**).

Although less prevalent, *P. zeae* exhibited a wide geographical distribution, occurring in all surveyed states except PI, where its absence is likely due to the limited sampling effort (only one sample analyzed). *Pratylenchus zeae* was the only species detected in three samples: UnBPratySoy42, UnBPratySoy83, and UnBPratySoy244. In 69 samples, *P*. *zeae* was found in association with *P. brachyurus*, and in one sample in association with both *P. brachyurus* and *P. penetrans* (UnBPratySoy272). *Pratylenchus zeae* was most frequent in GO (15 populations), followed by MT (14), RS (9), SC (7), MS (6), SP (5), ES (4), PR (3), MA, MG, and the DF (2 each), and AC, PA, BA, and TO (1 each).

*Pratylenchus brasiliensis* was detected in two populations from the present survey, originating from MA and TO. For comparative analyses, four additional populations previously reported by Diniz et al. (2026), from MT, TO, GO, and AC, were also included, resulting in six populations considered in total. In contrast, *P. penetrans* was rare, being detected in only one population from RS (**Figures 1A–E**; **Supplementary Table S1**).

### 3.2 Mixed-species populations of *Pratylenchus* in Brazil

Mixed-species populations were common and represented a significant component of *Pratylenchus* diversity in Brazilian soybean fields. A total of 72 of 296 populations contained more than one species (24%).

The combination of *P. brachyurus* and *P. zeae* was noteworthy, occurring in 70 mixed populations distributed across GO, MT, RS, SC, MS, SP, ES, DF, MA, MG, PR, AC, PA, BA, and TO. This constituted the principal pattern of species co-occurrence nationwide.

Two less frequent combinations were observed: one population from MA contained *P. brachyurus* and *P. brasiliensis*, and a single population from RS harbored three species, *P. brachyurus*, *P. zeae*, and *P. penetrans* (**Figures 1A–E**; **Supplementary Table S1**).

### 3.3 Phylogenetic relationships and variability of *P. brachyurus* and *P. zeae*

In total, 367 populations were analyzed and based on partial sequences of the ITS region, the D2–D3 expansion segments of the 28S rDNA gene, and of the mitochondrial COI gene four species were identified, *P. brachyurus*, *P. zeae*, *P. penetrans*, and *P. brasiliensis*. Phylogenetic analyses based on three molecular markers were conducted using reference sequences available in GenBank (**Supplementary Table S1**). All taxa formed strongly supported clades with reference specimens (posterior probability > 0.99; **Figures 2**-**4**).

Phylogenetic reconstructions revealed congruent clustering patterns across molecular markers, supporting the delimitation of *Pratylenchus* species. Particularly for *P. brachyurus* and *P. zeae*, analyses based on the 28S rDNA and ITS regions consistently recovered multiple well-supported lineages within each species, indicating the presence of genetically differentiated populations. In contrast, the mitochondrial COI gene provided a more cohesive grouping for most populations, showing reduced internal subdivision compared with nuclear markers.

The polytomy observed in *P. brachyurus* and *P. zeae* using ribosomal regions suggests the presence of substantial intraspecific genetic diversity. These findings highlight the importance of a multigene approach for accurate identification and phylogenetic interpretation of root-lesion nematodes associated with soybean (**Figures 2**-**4**).

### 3.4 Morphological and morphometric characterization of *Pratylenchus* populations associated with soybean fields in Brazil

Phylogenetic and morphological/morphometric analyses corroborated the occurrence of four distinct *Pratylenchus* species associated with soybean fields in Brazil, corresponding to *P. brachyurus*, *P. zeae*, *P. penetrans*, and *P. brasiliensis*. A total of 63 pure populations were evaluated, representing four morphotypes: 51 populations molecularly identified as *P. brachyurus*, nine as *P. zeae*, two as *P. brasiliensis*, and one as *P. penetrans*. Morphometric evaluations were performed on 10 adult females from each pure population. In addition, male specimens were measured when present, as observed in some populations of *P. brachyurus*, *P. penetrans*, and *P. brasiliensis*. No males were detected in any population of *P. zeae*. The morphometric and morphological data for diagnostic characters are summarized in **Table 1** and **Figures 5**–**8**, respectively.

**Figure 5:**
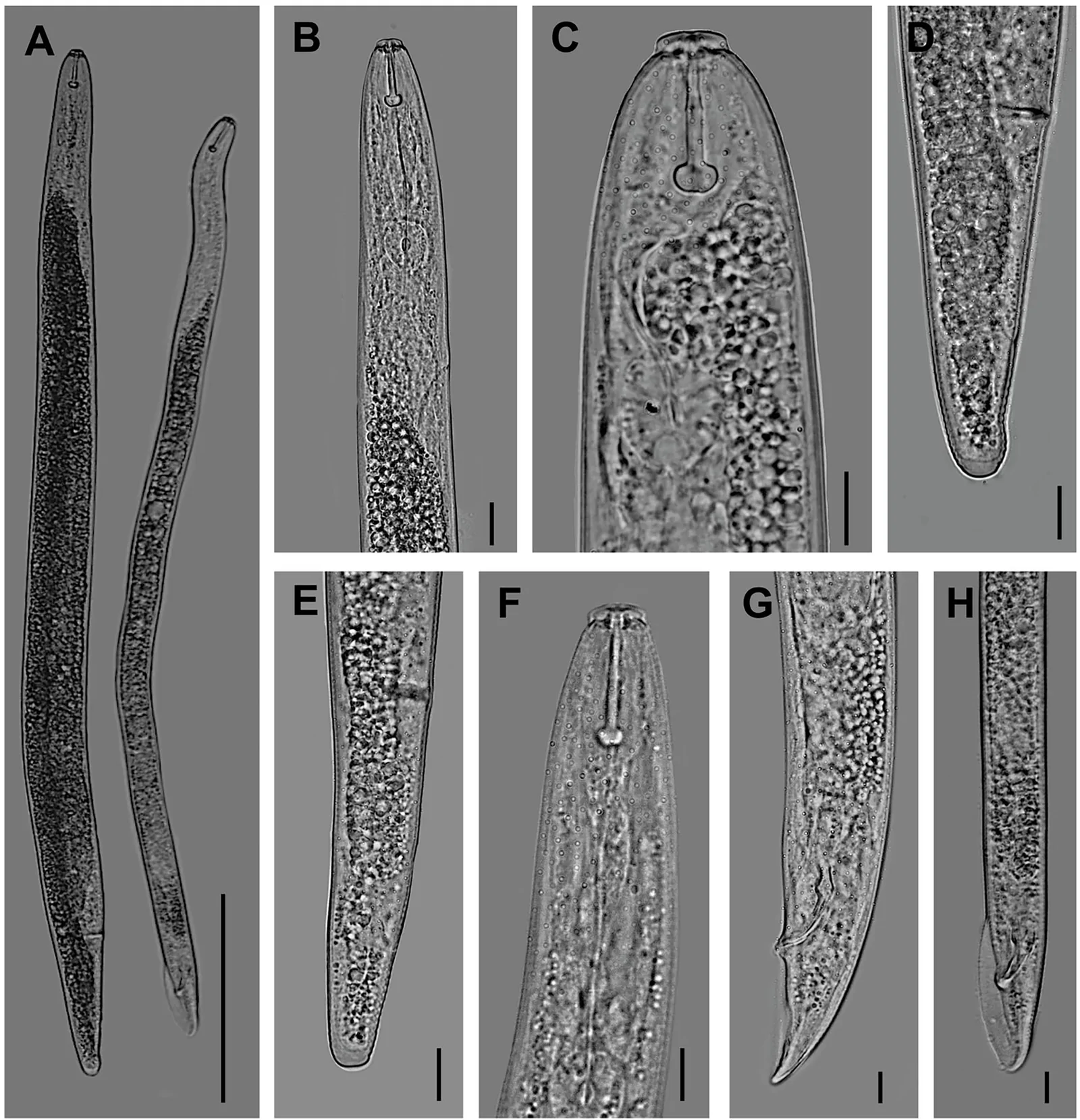
Morphological features of *Pratylenchus brachyurus*. Females **(A–E)**: **(A)** Whole body; **(B)** Anterior region showing smooth lip region with two annuli; **(C)** Stylet with rounded basal knobs; **(D, E)** Tail and detail of the tail showing a distinct hyaline region. Males **(A; F–H)**: **(F)** Whole body; **(G)** Anterior region with well-developed stylet; **(H)** Posterior region showing cloacal bursa, spicules, and gubernaculum. Morphometric data are provided in **Table 1**. Scale bars A: 100 µm and B-H: 10 µm.

**Figure 6:**
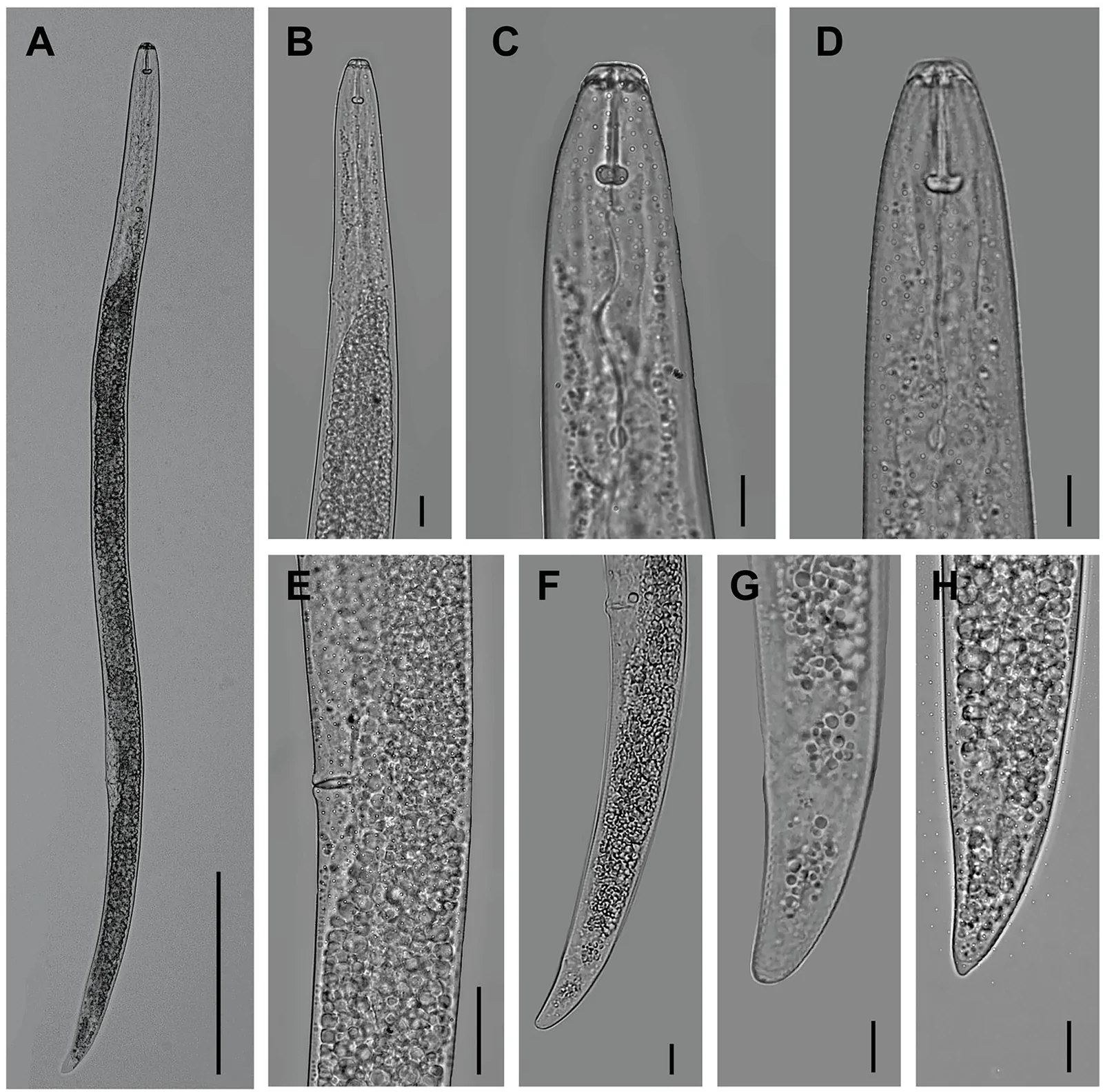
Morphological features of *Pratylenchus zeae*. Females **(A–H)**: **(A)** Whole body showing slender form with ventral curvature; **(B)** Anterior region; **(C, D)** Head region with smooth, continuous lip region; and stylet with broad, anchor-shaped or rounded basal knobs; **(E)** Vulval region; **(F)** Tail; **(G, H)** Detail of the conical tail tapering to a finely rounded terminus. No males were observed in the analyzed populations. Morphometric data are provided in Table. Scale bars A: 100 µm and B-H: 10 µm.

**Figure 7:**
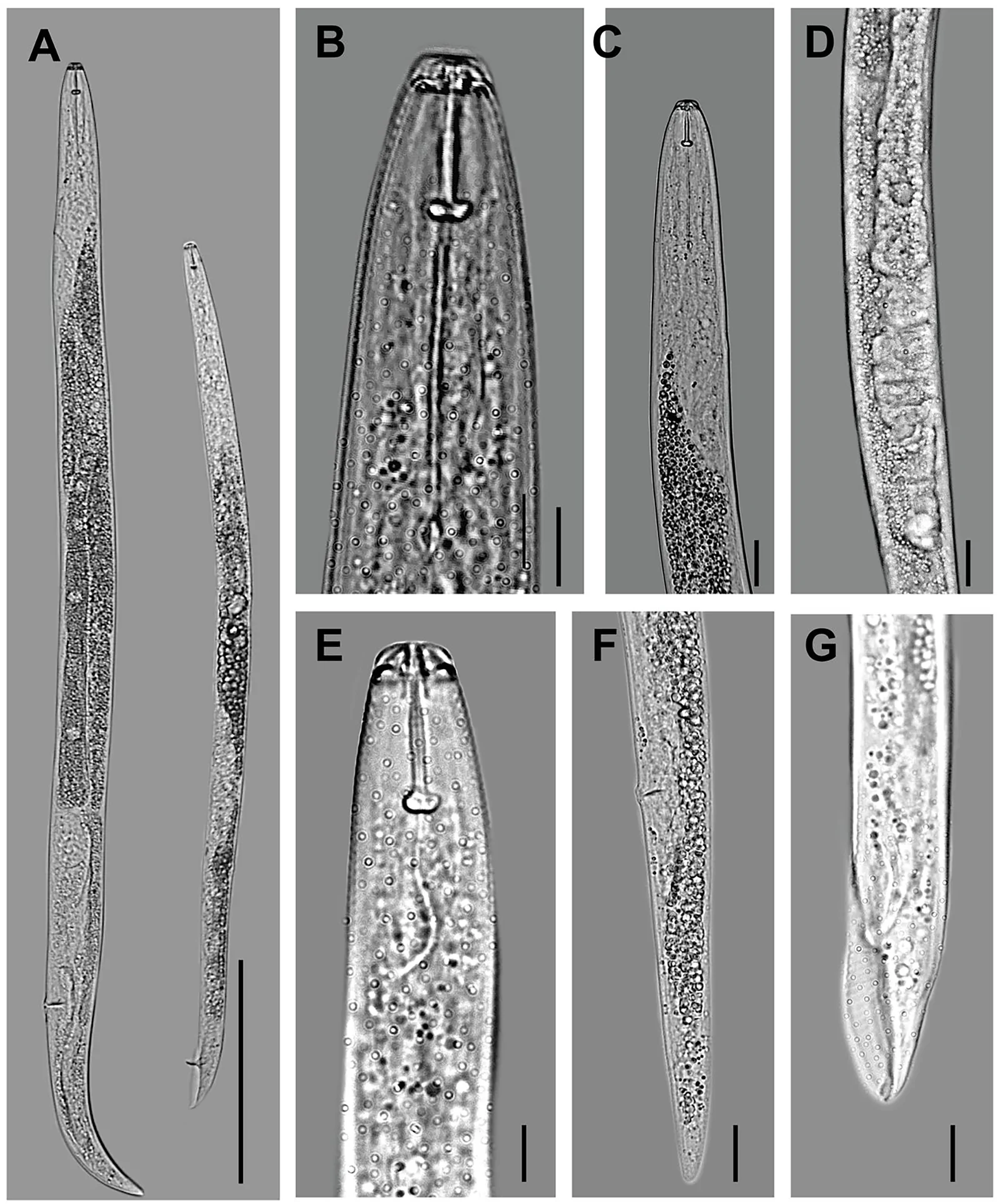
Morphological features of *Pratylenchus brasiliensis*. Females **(A–D, F)**: **(A)** Whole body; **(B)** Anterior region showing lip region with three-four annuli; **(C)** Stylet with rounded-shaped basal knobs; **(D)** Reproductive system showing the round spermatheca; **(F)** Tail and detail of the tail showing a short hyaline region. Males **(E, G)**: **(A)** Whole body; **(E)** Anterior region with well-developed stylet; and **(G)** Posterior region showing cloacal bursa, spicules, and gubernaculum. Morphometric data are provided in **Table 1**. Scale bars A: 100 µm and B-G: 10 µm.

**Figure 8:**
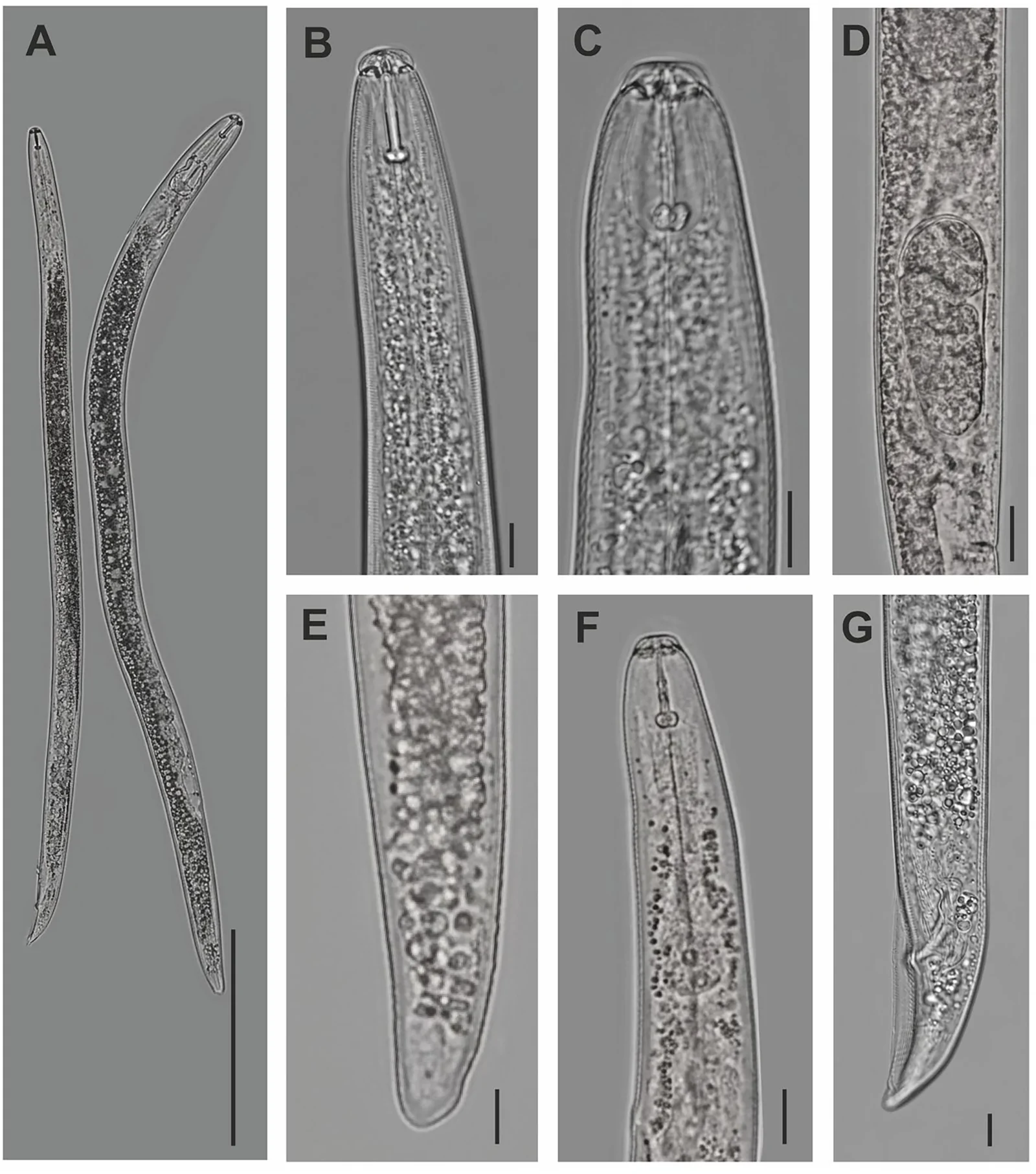
Morphological features of *Pratylenchus penetrans*. Females **(A–E)**: **(A)** Whole body; **(B)** Anterior region showing lip region; **(C)** Stylet with rounded-shaped basal knobs; **(D)** Reproductive system showing the round spermatheca; **(E)** Tail. Males **(A, F–G)**: **(A)** Whole body; **(F)** Anterior region with well-developed stylet; and **(G)** Posterior region showing cloacal bursa, spicules, and gubernaculum. Morphometric data are provided in **Table 1**. Scale bars A: 100 µm and B-G: 10 µm.

#### 3.4.1 Pratylenchus brachyurus

Specimens identified as *P. brachyurus* exhibited cylindrical and slightly curved bodies, with body length (L) ranging from 530.64 to 787.94 µm (mean = 649.9 ± 52.6 µm) and maximum body width (Øc) of 24.7–39.6 µm (**Figures 5A–E**). The lip region was continuous with the body contour, bearing two annuli, and the cuticle displayed fine transverse annulations (**Figures 5B, C**). The stylet (St) was well developed, measuring 19.4–22.5 µm (mean = 21.2 ± 0.7 µm), with rounded basal knobs 4.5–6.8 µm wide (ØbSt) and 2.9–4.2 µm high (AbSt) (**Figure 5C**). The distance from the anterior end to the excretory pore (DEA) varied from 90.9 to 128.0 µm (mean = 105.7 ± 7.2 µm), whereas the total esophageal length (CE) ranged from 140.5 to 175.7 µm (mean = 153.6 ± 7.3 µm). The vulva was positioned far posteriorly (V = 79.7–87.3%, mean = 85.7%), and the tail was conoid, smooth, and rounded to truncate, frequently exhibiting a distinct hyaline region (HC) 3.2–6.2 µm long (**Figures 5D, E**). Morphometric indices were a = 20.6 ± 1.6, b′ = 4.3 ± 0.3, and c = 19.8 ± 1.1 (**Figure 5**; **Table 1**).

Males of *P. brachyurus* were confirmed in 15 populations from Goiás (GO), Paraná (PR), Mato Grosso (MT), Mato Grosso do Sul (MS), Bahia (BA), São Paulo (SP), and Rio Grande do Sul (RS) (**Figures 5F–H**). Of these, seven populations contained sufficient males for morphometric analysis. When present, males occurred at low frequencies, typically at a ratio of approximately one male per 100 females (**Table 1**).

Male specimens showed morphology similar to females but were slightly shorter and more slender, with body length (L) ranging from 458.9 to 594.9 µm (mean = 519.8 ± 46.2 µm) and maximum body width (Øc) of 23.5–31.1 µm (mean = 26.6 ± 3.7 µm). The stylet (St) measured 16.9–20.9 µm (mean = 18.9 ± 1.1 µm), with rounded basal knobs 2.8–4.6 µm wide (ØbSt) and 2.8–4.1 µm high (AbSt) (**Figure 5F**). The distance from the anterior end to the excretory pore (DEA) ranged from 84.5 to 103.5 µm (mean = 94.9 ± 6.9 µm), and the total esophageal length (CE) varied between 118.0 and 146.9 µm (mean = 132.8 ± 8.7 µm). The pharyngeal gland overlap (PGO) measured 28.1–45.9 µm (mean = 34.3 ± 7.9 µm). Spicules measured 16.9–20.6 µm in length (Lspic) and 3.1–5.3 µm in width (Øspic). The bursa originated anterior to the cloaca and extended along the entire tail length (**Figures 5G, H**; **Table 1**).

#### 3.4.2 Pratylenchus zeae

Individuals molecularly identified as *P. zeae* exhibited slender bodies with a ventral curvature, with body length (L) ranging from 434.6 to 607.4 µm (mean = 511.2 ± 51.6 µm) (**Figures 6A–H**). The lip region was smooth and continuous, and the stylet (St) was comparatively shorter, measuring 15.4–17.2 µm (mean = 16.6 ± 0.6 µm), with broad, anchor-shaped or rounded basal knobs (**Figures 6B–D**). The distance from the anterior end to the excretory pore (DEA) measured 73.2–97.6 µm (mean = 81.5 ± 8.9 µm), whereas the total esophageal length (CE) ranged from 101.4 to 119.7 µm (mean = 114.2 ± 5.4 µm). The vulva was located at V = 70.4–73.3% of the body length (mean = 71.6%) (**Figures 6E, F**), and the tail was typically conical with a pointed terminus; however, variants with a rounded terminus and absence of striations were also observed (**Figures 6G, H**). Morphometric indices were a = 18.9 ± 1.6, b′ = 4.5 ± 0.3, and c = 17.8 ± 1.6. No males were observed in any of the pure populations examined (**Figure 6**; **Table 1**).

#### 3.4.3 Pratylenchus brasiliensis

A third morphotype was consistently recovered and molecularly identified as *P. brasiliensis* (**Figures 7A–G**). Females exhibited slightly curved bodies, with body length (L) ranging from 518.7 to 805.5 µm (mean = 637.8 ± 50.05 µm). The lip region was low and rounded, with lip diameter (ØL) of 8.45 ± 0.43 µm, and the stylet (St) was well developed, measuring 15.8–18.6 µm (mean = 17.1 ± 0.4 µm), bearing rounded basal knobs 3.2–4.8 µm wide (ØbSt) and 1.9–2.4 µm high (AbSt) (**Figures 7B, C**). The distance from the anterior end to the excretory pore (DEA) ranged from 89.6 to 113.2 µm (mean = 98.6 ± 5.4 µm), and the total esophageal length (CE) varied between 114.7 and 166.0 µm (mean = 137.9 ± 11.8 µm), with a distinct pharyngeal gland overlap.

The reproductive system was monodelphic–prodelphic, with a rounded spermatheca (**Figure 7D**). The vulva was positioned at V = 77.7–83.1% (mean = 79.9 ± 1.0%) of body length. The post-uterine sac (PUS) measured 29.3 ± 5.2 µm. The tail was conical, gradually tapering to a smooth, rounded terminus, with a short hyaline region (HC) (**Figure 7F**). Morphometric ratios were a = 22.5 ± 1.9, b′ = 4.7 ± 0.3, c = 19.2 ± 1.4, and c′ = 2.3 ± 0.17.

Males were frequently observed, occurring at an approximately 1:1 sex ratio with females. Males were smaller than females, with body length (L) of 474.4–538.3 µm (mean = 504.1 ± 20.5 µm). The lip region was similar in shape to that of females, and the stylet (St) was slightly shorter, measuring 15.5–16.8 µm (mean = 16.3 ± 0.4 µm), with rounded basal knobs. Spicules were well developed and arcuate, measuring 15.5–19.9 µm in length (Lspic) (mean = 18.2 ± 1.5 µm), accompanied by a distinct gubernaculum (gub). The bursa extended along the entire tail length. Morphometric indices for males were a = 25.3 ± 2.3, b′ = 4.1 ± 0.3, c = 19.0 ± 0.8, and c′ = 2.23 ± 0.18 (**Figure 7**; **Table 1**).

#### 3.4.4 Pratylenchus penetrans

The female isolates molecularly identified as *Pratylenchus penetrans* exhibited a slender body with ventral curvature, with body length (L) ranging from 625.28 to 779.17 µm (mean = 710.94 ± 57.09 µm) (**Figure 8A**). The lip region was rounded and continuous with the body contour (**Figures 8B, C**). The stylet (St) was robust, measuring 16.20–18.73 µm (mean = 17.76 ± 0.74 µm), with basal knobs varying from rounded to anteriorly concave (**Figure 8C**). The distance from the anterior end to the excretory pore (DEA) ranged from 91.35 to 111.60 µm (mean = 102.98 ± 7.53 µm), while the total esophagus length (CE) varied from 122.82 to 160.53 µm (mean = 143.27 ± 13.66 µm). The vulva was located at V = 78.22–81.64% of the body length (mean = 79.53%) (**Figures 8A, D**). The tail was slightly cylindrical, measuring 34.01–42.68 µm (mean = 37.82 ± 3.06 µm), with a smooth terminus (**Figures 8A, E**). The morphometric ratios were a = 22.24 ± 2.26, b′ = 4.93 ± 0.33, c = 18.81 ± 0.79, and c’ = 2.00 ± 0.17.

Males were commonly observed and were morphologically similar to females in all non-sexual characters but smaller in size, with body length (L) ranging from 613.70 to 663.17 µm (mean = 635.68 ± 16.68 µm) (**Figure 8A**) and maximum body width (Øc) of 22.08–24.57 µm (mean = 23.39 ± 0.83 µm). The stylet (St) measured 14.05–16.96 µm (mean = 16.06 ± 1.03 µm), with rounded, weakly developed basal knobs, wider than high (**Figure 8F**). The distance from the anterior end to the excretory pore (DEA) ranged from 89.55 to 106.47 µm (mean = 98.55 ± 5.54 µm), and the total esophagus length (CE) varied from 115.37 to 136.18 µm (mean = 129.78 ± 8.38 µm). The pharyngeal gland overlap (PGO) measured 22.01–35.70 µm (mean = 29.68 ± 5.32 µm). Spicules measured 15.50–19.61 µm in length (Lspic) and 1.61–3.48 µm in width (Øspic). The bursa originated anterior to the cloaca and extended along the entire length of the tail (**Figure 8G**; **Table 1**).

### 3.5 PCA and comparative morphometric and morphological analysis among *Pratylenchus* species associated with soybean fields

PCA of the morphometric dataset did not separate among *P. zeae*, *P. brasiliensis*, and *P. penetrans* associated with soybean in this study, nor between these populations and previously reported isolates of *P. penetrans* (Janssen et al., 2017; Gil et al., 2021) and *P. brasiliensis* (Diniz et al., 2026) (**Figure 9**). This pattern was further supported by the substantial overlap observed among *P. zeae*, *P. penetrans*, and *P. brasiliensis*, highlighting pronounced intraspecific morphometric variability and the limited resolution of quantitative traits when used alone. In contrast, *P. brachyurus* formed a more distinct cluster, as indicated by the 95% confidence ellipses.

**Figure 9:**
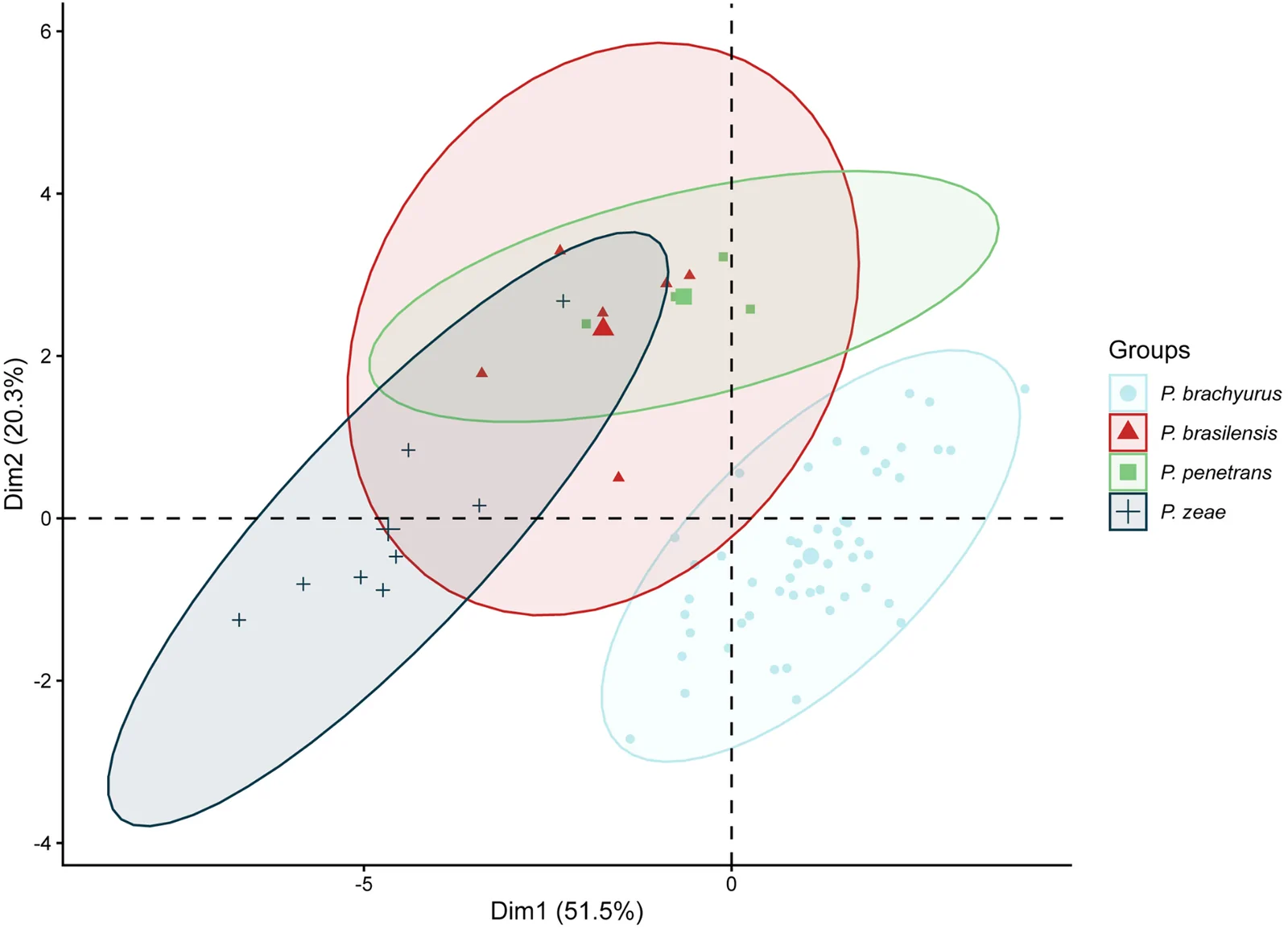
Principal Component Analysis (PCA) based on morphometric data of four *Pratylenchus* species (*Pratylenchus brachyurus, P. brasilensis, P. penetrans* and *P. zeae*). The PCA was constructed using the following variables: body length (L), vulva position (V), morphometric ratios *a*, *b’* and *c*, stylet length (St), post-uterine sac length (PUS), distance from the anterior end to the excretory pore (DEA), esophagus length (CE), mean body diameter (Øc), and tail length (CT). The first two components explain 51.5% (Dim1) and 20.3% (Dim2) of the total variance. Points represent individual specimens, colors and symbols indicate species, and ellipses correspond to 95% confidence regions, highlighting the morphometric separation among the analyzed species.

The first two principal components explained 71.8% of the total variance, with Dim1 accounting for 51.5% and Dim2 for 20.3%. Dim1 was primarily influenced by body size (L) and structural traits, including esophagus length (CE; 17.02%), distance from the anterior end to the excretory pore (DEA; 17.01%), stylet length (St; 13.31%), vulva position (V; 14.87%), and body length (L; 15.7%). Dim2 was mainly associated with the morphometric ratios b′ (40.65%) and a (20.90%), highlighting the contribution of proportional to secondary differentiation among species.

Comparative morphological analyses were consistent with the multivariate patterns observed in the PCA; however, species-level discrimination within *Pratylenchus* remained challenging, particularly for distinguishing *P. brasiliensis* from *P. penetrans*. St and basal knob morphology were among the most diagnostic characters: *P. brachyurus* had the longest stylet (mean = 21.2 µm), with robust, rounded basal knobs, whereas *P. zeae* had the shortest stylet (mean = 16.6 µm), with wide, anchor-shaped or rounded knobs. *Pratylenchus brasiliensis* and *P. penetrans* exhibited intermediate stylet lengths and similar rounded basal knobs. The absence of males in the *P. zeae* populations examined also provided an additional diagnostic feature that aided its differentiation from the other species evaluated.

Additional interspecific differences were observed in the distance to the DEA, CE, and V%. *Pratylenchus brachyurus* consistently showed the highest mean values for DEA and CE, whereas *P. zeae* had the lowest. The vulva position was more posterior in *P. brachyurus* (mean V = 85.7%), markedly more anterior in *P. zeae* (mean V = 71.6%), and intermediate in *P. brasiliensis* and *P. penetrans* (~79%). Males were absent in *P. zeae*, rare in *P. brachyurus*, and consistently present at approximately a 1:1 ratio in *P. penetrans* and *P. brasiliensis*.

Tail morphology was highly variable both within and among species and did not allow reliable discrimination. Multiple tail shapes were observed within the same species, and similar morphotypes were shared across species, resulting in extensive overlap and preventing separation based solely on this character.

The partial overlap among species and the absence of well-defined clusters for each *Pratylenchus* species, particularly for *P. penetrans* and *P. brasiliensis*, indicate that morphometric data alone are insufficient for reliable species discrimination and should be complemented with molecular analyses.

### 3.6 Interaction and reproduction of *Pratylenchus-*soybean

ANOVA revealed a significant effect of soybean cultivar on the Rf of both *P. brasiliensis* and *P. brachyurus* in Experiment 1 (*P. brasiliensis*: *F*(7, 48) = 4.79, *p* = 0.00038; *P. brachyurus*: *F*(7, 48) = 10.88, *p* = 3.97 × 10^-8^). In Experiment 2, soybean cultivar also significantly affected the Rf of both *P. brachyurus* (*F*(7, 40) = 9.95, *p* = 4.00 × 10^-7^) and *P. brasiliensis* (*F*(7, 40) = 2.22, *p* = 0.05).

Across the evaluated cultivars, *P. zeae* showed relatively low Rf values, ranging from 0.06 to 0.27 in experiment 1 and from 0.17 to 0.33 in experiment 2, indicating poor host suitability for soybean. In contrast, *P. brasiliensis* and *P. brachyurus* exhibited comparatively high Rf values across the evaluated cultivars, ranging from 4.94 to 7.63 and 3.33 to 8.17 in experiment 1, and from 4.66 to 10.84 and 4.93 to 11.44 in experiment 2, respectively (**Figure 10**).

**Figure 10:**
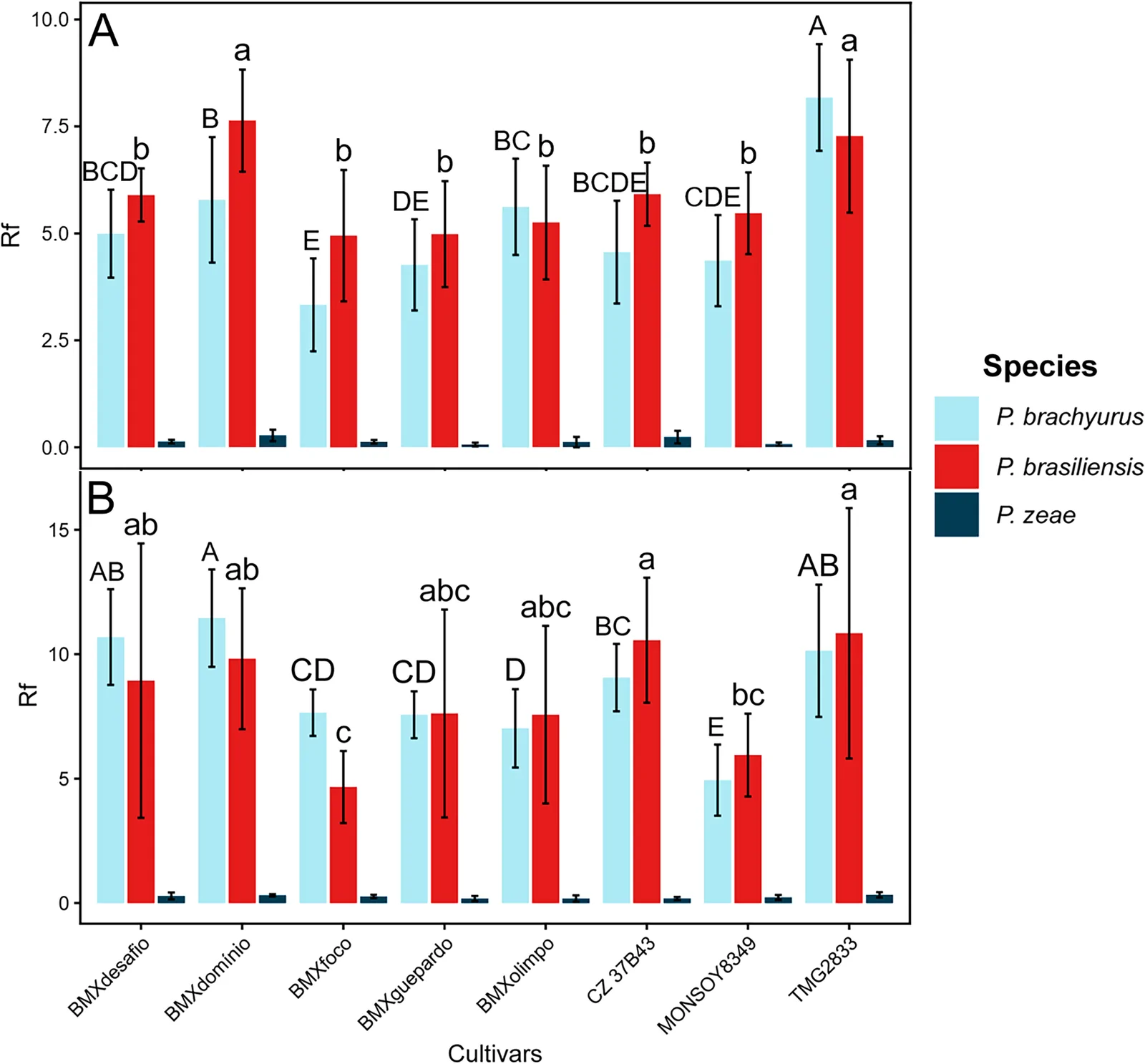
Reproduction factor (Rf) of *Pratylenchus zeae*, *P. brasiliensis* and *P. brachyurus* on soybean cultivars in two greenhouse experiments. **(A)** First experiment. **(B)** Second experiment. Bars represent mean Rf values; different letters indicate significant differences among cultivars within each species (*P. brasiliensis* and *P. brachyurus*) according to Fisher’s LSD test (p ≤ 0.05). Different lowercase letters indicate significant differences among soybean cultivars within each *Pratylenchus* species, whereas different uppercase letters indicate significant differences among *Pratylenchus* species within each soybean cultivar, based on Fisher’s LSD test at the 5% significance level. An Rf value greater than 1 indicates susceptibility of cultivars.

For *P. brasiliensis*, significant cultivar-dependent differences were detected in both experiments (p< 0.001). In the first experiment, the highest Rf values were observed in ‘BMX Domínio’ and ‘TMG 2833’ (**Figure 10A**). In the second experiment, ‘TMG 2833’ and ‘CZ 37B43’ exhibited the highest Rf values, followed by ‘BMX Domínio’ and ‘BMX Desafio’, while ‘BMX Foco’, ‘MONSOY 8349’ and ‘BMX Olimpo’ showed reduced reproduction relative to the other cultivars (**Figure 10B**). Despite these differences, all cultivars displayed Rf values greater than 1, indicating susceptibility to *P. brasiliensis*.

A similar pattern was observed for *P. brachyurus* in the first and second experiment, with significant differences among cultivars in both experiments (p< 0.001). ‘TMG 2833’, ‘BMX Domínio’ and ‘BMX Desafio’ consistently supported the highest nematode reproduction and were identified as the most susceptible cultivars. In contrast, ‘BMX Foco’ and ‘MONSOY 8349’ exhibited comparatively lower Rf values, yet still allowing successful nematode reproduction. Rf values exceeded 1 for all cultivars, demonstrating the absence of effective resistance to *P. brachyurus* across the assessed soybean genotypes (**Figures 10A, B**).

## 4 Discussion

This is the most comprehensive survey and polyphasic identification study of *Pratylenchus* associated with soybean in Brazil. The study integrates large-scale field sampling, detailed morphological and morphometric characterization, multilocus phylogenetic analyses, and assessment of resistance of soybean cultivars to *Pratylenchus* spp. Species of *Pratylenchus* were pervasive in Brazilian soybean fields. Mixed infestations occurred roughly in one out of four samples. A total of 72 samples contained mixed populations comprising two or more *Pratylenchus* species. Among these, 71 samples harbored two species, whereas one sample contained a mixture of three species: *P. brachyurus*, *P. zeae*, and *P. penetrans*. *Pratylenchus brachyurus* and *P. zeae* were the prevalent species with vast distribution in the country and exhibited high genetic variability, with divergent lineages. This study also provides the first confirmed association of *P. penetrans* with soybean in Brazil and documents the presence of males in *P. brachyurus* populations from multiple Brazilian states. In addition, the results highlight the persistent difficulty in differentiating *P. penetrans* and *P. brasiliensis* using morphology alone, reinforcing the need for integrative taxonomic approaches to accurately resolve species boundaries within the genus. The widespread occurrence of *Pratylenchus* across Brazilian soybean fields underscores the difficulty of controlling these nematodes, particularly given the lack of resistance in major soybean cultivars to *P. brachyurus* and *P. brasiliensis*. Although *P. zeae* commonly penetrates soybean roots, its populations remain low and are likely sustained by continuous soybean–corn succession, which enables population carryover between seasons.

Among the four species detected, *P. brachyurus* clearly emerged as the most prevalent and geographically widespread taxon, occurring in virtually all soybean-producing regions and in nearly every positive sample analyzed. This pattern corroborates previous regional surveys in Brazil, which consistently identified *P. brachyurus* as the prevalent root-lesion nematode in soybean fields across different states and production systems (Ribeiro et al., 2013). Its occurrence across contrasting climates, soils, cropping histories, and even areas of natural vegetation suggests broad ecological adaptability and a long-standing association with Brazilian agroecosystems and native environments (Goulart et al., 2003).Together with the genetic variability observed among Brazilian populations, these findings raise the hypothesis that Brazil, or more broadly South America, could represent an important region in the evolutionary history and diversification of *P. brachyurus*.

The detection of *P. penetrans* in soybean fields in RS is the first confirmed report of this species parasitizing soybean in Brazil. Its occurrence was geographically restricted and detected at low frequency, consistent with previous sparse records of this species in southern and southeastern Brazil (Ferraz and Monteiro, 1983; Monteiro and Covolo, 1985; Rossi and Monteiro, 2001; Oliveira et al., 2009). This restricted distribution is also compatible with the temperate adaptation of *P. penetrans*, whose development is reduced at higher temperatures (Mizukubo and Adachi, 1997). This pattern may help explain its apparent confinement to cooler soybean-producing regions in southern Brazil.

In contrast, two additional isolates of *P. brasiliensis*, a species recently described, were detected across soybean-producing areas in northern and northeastern Brazil. Although detected at low frequency, its occurrence in tropical soybean systems suggests that *P. brasiliensis* may be more widely distributed than currently recognized, particularly in regions where nematological surveys remain scarce (Diniz et al., 2026). This finding underscores the importance of broad-scale surveys for accurately characterizing nematode diversity in agroecosystems, rather than providing evidence for host shifts or disease emergence.

Mixed *Pratylenchus* populations were detected in 24% of the sampled fields (72 samples). The predominance of *P. brachyurus* and *P. zeae* indicates that soybean roots in Brazil are frequently exposed to simultaneous infection by multiple root-lesion nematode species. This pattern may be driven by the widespread adoption of soybean–corn rotation systems, which can favor the persistence of both taxa across seasons: *P. brachyurus* is preferentially associated with dicotyledonous hosts, whereas *P. zeae* is more commonly linked to monocotyledonous crops (Rossi et al., 2000; Franchini et al., 2018; Santana-Gomes et al., 2019). Accordingly, *P. zeae* is a major constraint in sugarcane, corn, and other grasses, while *P. brachyurus* is particularly damaging to dicot crops, including soybean (Dinardo-Miranda et al., 2019; Franchini et al., 2018; Santana-Gomes et al., 2019). These findings demonstrate that soybean roots in Brazil are frequently exposed to simultaneous infection by multiple lesion nematode species, challenging the traditional single-species framework that underlies most diagnostic, breeding strategies, and management approaches.

Although *P. zeae* was able to penetrate soybean roots and persist at low population levels, susceptibility assays demonstrated that its reproduction was insufficient to maintain or increase the initial inoculated population, with Rf values consistently below 1 across all evaluated cultivars, indicating resistance. The recurrent presence of *P. zeae* in soybean fields, probably driven by the commonly adopted soybean–corn rotation system, enables population carryover across cropping seasons. Such mixed infestations may intensify root damage and shift yield-loss patterns, thus complicating diagnosis and reducing the effectiveness of species-specific management strategies (Smiley et al., 2014; Begum et al., 2019; Copeland et al., 2025).

All major commercial soybean cultivars grown in Brazil were susceptible to *P. brachyurus* and *P. brasiliensis*, reinforcing their importance as root-lesion nematodes associated with soybean. Notably, cultivar response was strongly species-dependent, with substantial variation in reproductive success among cultivars, such that individual genotypes differentially favored multiplication of *P. brachyurus* or *P. brasiliensis*. These results suggest that susceptibility is better interpreted as a quantitative response shaped by specific host–nematode interactions rather than as a strictly binary trait. However, because only one population of each *Pratylenchus* species was evaluated, the observed cultivar-response patterns should not be generalized to the species level. Intraspecific variation among nematode populations may influence reproductive performance and host response, and this remains to be tested using a broader set of geographically and genetically distinct populations. While distinct patterns were observed among the *Pratylenchus* species evaluated, additional experiments including multiple populations per species are therefore required to determine the robustness and consistency of these responses across the genetic diversity of each species. Nevertheless, accurate *Pratylenchus* species identification remains an important component of cultivar selection and nematode management.

At a broader geographic scale, the composition of *Pratylenchus* communities associated with soybean differs markedly worldwide. In North America, *P. penetrans* and *P. neglectus* are among the most frequently reported lesion nematodes affecting soybean and other temperate crops, reflecting adaptation to cooler environments and diverse crop rotations (Riggs, 1992; Bélair et al., 2018; Powers et al., 2021). In East and Southeast Asia, soybean-associated *Pratylenchus* communities commonly include *P. coffeae* and *P. loosi*, reflecting regional cropping systems and climatic conditions (Tuyet, 2010). In Brazil, *P. brachyurus* is prevalent in soybean fields, occurring either alone or in mixed populations with *P. zeae* and, in North/Northeast regions of Brazil, *P. brasiliensis*, all of which are well adapted to warm climates and intensive soybean production systems. These patterns highlight the influence of climate, species origin, and cropping history on *Pratylenchus* assemblages and underscore the need for region specific diagnostics and management strategies rather than generalized control recommendations.

One important finding of this study is the exceptionally high genetic variability observed among Brazilian populations of *P. brachyurus* and *P. zeae*. Multilocus phylogenetic analyses revealed multiple well-supported evolutionary lineages within each species, resulting in polytomic patterns with consistent species identification. The high variability is unexpected for taxa reported to reproduce predominantly by parthenogenesis, as described for *P. brachyurus* and *P. zeae*. In asexual or quasi asexual systems, the accumulation of mutations in the absence of recombination can lead to deep lineage divergence, which likely explains the coexistence of genetically distinct intraspecific clades occurring sympatrically and even within the same soybean field (Lovell et al., 2017; Villegas et al., 2024).

The detection of males in Brazilian populations of *P. brachyurus*, reported here for the first time, does not indicate a novel biological trait, but rather reflects a historically rare and previously undocumented character, likely overlooked due to under sampling in Brazil. For example, males of *P. brachyurus* were detected in North America and Africa (Castillo and Vovlas, 2007). Males occurred at low frequency in Brazil, approximately one male per 100 females, suggesting that reproduction is predominantly asexual, with the potential for rare or facultative sexual events. Although the reproductive functionality of these males remains unknown, their occurrence highlights a poorly understood aspect of the reproductive biology of *P. brachyurus* that warrants further investigation. Independently of the occurrence of males, the molecular and phylogenetic variation detected among Brazilian populations indicates that *P. brachyurus* should not be regarded as a genetically uniform pathogen but rather as a species comprising genetically differentiated lineages.

The morphological and morphometric characteristics of Brazilian populations of *P. brachyurus* and *P. zeae* were largely consistent with classical species descriptions, supporting their correct identification in soybean producing areas. Females and males of *P. brachyurus* matched the original descriptions by Godfrey (1929), while Brazilian populations of *P. zeae*, *P. penetrans*, and *P. brasiliensis* were consistent with the descriptions by Graham (1951); Cobb (1917) Filipjev and Schuurmans Stekhoven, 1941; and Diniz et al. (2026), respectively. These results confirm the occurrence of four *Pratylenchus* species in Brazilian soybean fields and validate the use of classical morphological traits for initial species assignment. At the same time, high levels of morphological and morphometric variability were observed among populations, complementing the extensive genetic diversity detected in multilocus analyses. Multivariate morphometric analysis clearly separated *P. brachyurus* and *P. zeae*, reinforcing the diagnostic value of phenotype-based approaches for rapid screening, particularly where molecular tools are not readily available.

The main species found in this study markedly differed regarding morphological traits. *Pratylenchus brachyurus* is distinguished by a robust stylet with spherical basal knobs and a posterior vulva position, whereas *P. zeae* exhibits a less robust stylet with anchor-shaped basal knobs and a more anterior vulva position. In contrast, the separation of *P. penetrans* and *P. brasiliensis* using morphology and morphometrics alone remains problematic. Both species commonly present males, share similar stylet base morphology, and display overlapping vulval position values, with reliable differentiation often requiring subtle features of the facial region observable only under transmission electron microscopy. This morphological overlapping, together with the high intraspecific variability documented here and the limited availability of specialized nematode taxonomists in Brazil, likely explain the longstanding difficulty in distinguishing *P. penetrans* from *P. brasiliensis* based solely on classical diagnostic characters.

These findings reveal that *Pratylenchus* populations associated with soybean in Brazil are far more diverse and evolutionarily complex than previously recognized. This complexity has direct implications for nematode diagnostics, resistance screening, and management strategies, highlighting the need for integrative, region-specific approaches that account for mixed infestations and intraspecific variation. Future studies incorporating broader global sampling, genome-wide markers, variability studies, and host interaction assays will be essential to fully resolve species boundaries, clarify evolutionary relationships, and improve the durability of nematode management strategies in soybean production systems.

## Data Availability

The datasets presented in this study can be found in online repositories. The names of the repository/repositories and accession number(s) can be found in the article/**Supplementary Material**.
