# Supplementary material for: Pratylenchus brachyurus is the prevalent root-lesion nematode in brazilian soybean fields, occurring singly and in mixed populations with P. zeae, P. penetrans, and P. brasiliensis
Source: Front Plant Sci. 2026 Oct 8;17:1951549. doi: 10.3389/fpls.2026.1951549 (PMC13687066; doi:10.3389/fpls.2026.1951549)
Supplement: Supplementary file 1 [file Table1.docx]

Supplementary Material

# 1 Supplementary Data

**Supplementary Table 1.** Identification and geographic distribution of *Pratylenchus* populations collected from soybean fields across five Brazilian macroregions and 16 states: North (Acre [AC], Pará [PA], and Tocantins [TO]), Northeast (Bahia [BA], Maranhão [MA], and Piauí [PI]), Central-West (Federal District [DF], Goiás [GO], Mato Grosso [MT], and Mato Grosso do Sul [MS]), Southeast (Minas Gerais [MG], São Paulo [SP], and Espírito Santo [ES]), and South (Paraná [PR], Santa Catarina [SC], and Rio Grande do Sul [RS]), between January 2021 and March 2023, based on phylogenetic analyses of the D2–D3 expansion segments of 28S rDNA, internal transcribed spacer (ITS) rRNA, and partial mitochondrial cytochrome c oxidase subunit I (COI). GenBank accession numbers for all sequences are provided. An asterisk (*) indicates populations in which males of *Pratylenchus brachyurus* were observed.

| **Samples** | **Phylogeny Identification** | **Municipality** | **State** | **Longitude** | **Latitude** | **GenBank 28s** | **GenBank ITS** | **GenBank COI** |
| --- | --- | --- | --- | --- | --- | --- | --- | --- |
| UnBPratySoy01 | *Pratylenchus brachyurus* | Brasiléia | AC | -68,809466 | -11,06569 | PZ662382 |  | PZ664873 |
| UnBPratySoy02 | *P. brachyurus* and *P. zeae* | Capixaba | AC | -67,684413 | -10,586968 | PZ487348 and PZ487255 | PZ483958 and PZ670761 | PZ473217 |
| UnBPratySoy03 | *P. brachyurus* and *P. zeae* | Dom Elizeu | PA | -47,520893 | -4,7520893 | PZ487237 |  | PZ473221 |
| UnBPratySoy04 | *P. brachyurus* | Baixa Grande do Ribeiro | PI | -45,169666 | -7,903539 | PZ487517 | PZ484148 | PZ473306 |
| UnBPratySo05 | *P. brachyurus* | Balsas | MA | -46,167263 | -7,535931 | PZ487516 | PZ484147 | PZ473308 |
| UnBPratySoy06 | *P. brachyurus* and *P. brasiliensis* | Balsas | MA | -46,31827 | -7,57634 | PZ487515 and PZ483001 | PZ484146 and PZ483765 | PZ464717 |
| UnBPratySoy07 | *P. brachyurus* and *P. zeae* | Igarapé | MA | -45,191223 | -3,64849 | PZ487338 and PZ487246 | PZ483951 and PZ483770 |  |
| UnBPratySoy08 | *P. brachyurus* and *P. zeae* | Itapecuru | MA | -44,360089 | -3,377433 | PZ487342 and PZ487257 | PZ483952 and PZ483799 |  |
| UnBPratySoy09 | *P. brasiliensis* | Silvanópolis | TO | -48,174183 | -11,158331 | PZ483000 |  | PZ464718 |
| UnBPratySoy10 | *P. brachyurus* and  *P. zeae* | Itapiratins | TO | -48,099518 | -8,343613 | PZ487327 and PZ487245 | PZ483936 and PZ483779 | PZ473207 |
| UnBPratySoy11 | *P. brachyurus* | Correntina | BA | -46,1985353 | -13,9519613 | PZ487365 | PZ483976 |  |
| UnBPratySoy12 | *P. brachyurus* | Correntina | BA | -46,1985353 | -13,9519613 | PZ487366 | PZ483977 | PZ473224 |
| UnBPratySoy13 | *P. brachyurus* | Correntina | BA | -46,1985353 | -13,9519613 | PZ487376 |  |  |
| UnBPratySoy14 | *P. brachyurus* | Jaborandi | BA | -44,467248 | -13,624149 | PZ487456 | PZ484075 |  |
| UnBPratySoy15 | *P. brachyurus* | Jaborandi | BA | -44,467248 | -13,624149 | PZ487457 | PZ484076 | PZ473276 |
| UnBPratySoy16 | *P. brachyurus* | São Desidério | BA | -44,97843 | -12,360721 | PZ487367 |  | PZ473225 |
| UnBPratySoy17 | *P. brachyurus* | São Desidério | BA | -44,97843 | -12,360721 |  | PZ483978 |  |
| UnBPratySoy18 | *P. brachyurus* | São Desidério | BA | -44,97843 | -12,360721 | PZ487368 | PZ483979 |  |
| UnBPratySoy19 | *P. brachyurus* | São Desidério | BA | -44,97843 | -12,360721 | PZ487369 | PZ483980 |  |
| UnBPratySoy20 | *P. brachyurus* | São Desidério | BA | -44,97843 | -12,360721 |  | PZ483981 |  |
| UnBPratySoy21 | *P. brachyurus* | São Desidério | BA | -44,97843 | -12,360721 |  | PZ483982 |  |
| UnBPratySoy22 | *P. brachyurus* | São Desidério | BA | -44,97843 | -12,360721 | PZ487370 | PZ483983 |  |
| UnBPratySoy23 | *P. brachyurus* | São Desidério | BA | -44,97843 | -12,360721 | PZ487371 | PZ483984 |  |
| UnBPratySoy24 | *P. brachyurus* | São Desidério | BA | -44,97843 | -12,360721 | PZ487372 | PZ483985 | PZ473226 |
| UnBPratySoy25 | *P. brachyurus* | São Desidério | BA | -44,97843 | -12,360721 | PZ487373 | PZ483986 | PZ473227 |
| UnBPratySoy26 | *P. brachyurus* | São Desidério | BA | -44,97843 | -12,360721 | PZ487374 | PZ483987 | PZ473228 |
| UnBPratySoy27 | *P. brachyurus* | São Desidério | BA | -44,97843 | -12,360721 | PZ487375 | PZ483988 |  |
| UnBPratySoy28 | *P. brachyurus* | São Desidério | BA | -44,97843 | -12,360721 | PZ487377 | PZ483989 | PZ473229 |
| UnBPratySoy29 | *P. brachyurus* and *P. zeae* | São Desidério | BA | -44,97843 | -12,360721 | PZ487378 and PZ671228 | PZ483990 and PZ483796 | PZ473230 |
| UnBPratySoy30 | *P. brachyurus* | São Desidério | BA | -44,97843 | -12,360721 | PZ487379 | PZ483991 |  |
| UnBPratySoy31* | *P. brachyurus* | Luiz Eduardo Magalhães | BA | -45,8538889 | -12,0727778 | PZ487333 | PZ483942 |  |
| UnBPratySoy32 | *P. brachyurus* | Luiz Eduardo Magalhães | BA | -45,8538889 | -12,0727778 | PZ487334 | PZ483943 |  |
| UnBPratySoy33 | *P. brachyurus* | Luiz Eduardo Magalhães | BA | -45,8538889 | -12,0727778 |  | PZ483944 |  |
| UnBPratySoy34 | *P. brachyurus* | Luiz Eduardo Magalhães | BA | -45,8538889 | -12,0727778 | PZ487512 | PZ484143 |  |
| UnBPratySoy35 | *P. brachyurus* | Riachão das Neves | BA | -45,715931 | -11,684198 | PZ487335 | PZ483945 | PZ473210 |
| UnBPratySoy36 | *P. brachyurus* | Riachão das Neves | BA | -45,715931 | -11,684198 | PZ487336 | PZ483946 | PZ473211 |
| UnBPratySoy37 | *P. brachyurus* | Riachão das Neves | BA | -45,715931 | -11,684198 | PZ487337 | PZ483947 | PZ473212 |
| UnBPratySoy38 | *P. brachyurus* | Riachão das Neves | BA | -45,715931 | -11,684198 | PZ487339 | PZ483948 | PZ473213 |
| UnBPratySoy39 | *P. brachyurus* | Riachão das Neves | BA | -45,715931 | -11,684198 | PZ487340 | PZ483949 | PZ473214 |
| UnBPratySoy40 | *P. brachyurus* and *P. zeae* | Barra do Garça | MT | -52,295291 | -15,919937 | PZ487321 and PZ487247 | PZ483928 and PZ483789 | PZ473202 |
| UnBPratySoy41 | *P. brachyurus* | Primavera do Leste | MT | -54,316271 | -15,507067 | PZ487452 | PZ484071 |  |
| UnBPratySoy42 | *P. zeae* | Barra dos Bugres | MT | -57,189032 | -15,074678 | PZ671225 | PZ483800 |  |
| UnBPratySoy43 | *P. brachyurus* | Barra do Bugres | MT | -57,189032 | -15,074678 |  | PZ484074 |  |
| UnBPratySoy44 | *P. brachyurus* | Diamantino | MT | -56,372712 | -14,39849 | PZ487355 | PZ483966 |  |
| UnBPratySoy45 | *P. brachyurus* | Campo novo do Parecis | MT | -57,82411 | -13,745479 | PZ487453 | PZ484072 | PZ473274 |
| UnBPratySoy46 | *P. brachyurus* | Lucas do Rio Verde | MT | -55,9811111 | -13,7336111 | PZ487270 | PZ483881 | PZ473165 |
| UnBPratySoy47 | *P. brachyurus* and *P. zeae* | Lucas do Rio Verde | MT | -55,9811111 | -13,7336111 | PZ487273 and PZ487263 | PZ670767 |  |
| UnBPratySoy48 | *P. brachyurus* | Lucas do Rio Verde | MT | -55,9811111 | -13,7336111 | PZ487285 | PZ483891 | PZ473176 and PZ473164 |
| UnBPratySoy49 | *P. brachyurus* and *P. zeae* | Nova Mutum | MT | -56,1696944 | -13,57475 | PZ487269 and PZ487266 | PZ483886 and PZ483791 |  |
| UnBPratySoy50 | *P. brachyurus* and *P. zeae* | Nova Mutum | MT | -56,1696944 | -13,57475 | PZ487271 and PZ487265 | PZ483882 and PZ670764 |  |
| UnBPratySoy51* | *P. brachyurus* | Nova Mutum | MT | -56,1696944 | -13,57475 | PZ487272 | PZ483884 | PZ473166 |
| UnBPratySoy52 | *P. brachyurus* and *P. zeae* | Nova Mutum | MT | -56,1696944 | -13,57475 | PZ487274 and PZ487264 | PZ483883 | PZ473167 |
| UnBPratySoy53 | *P. brachyuru*s and *P. zeae* | Nova Mutum | MT | -56,1696944 | -13,57475 | PZ487277 | PZ670766 and PZ670762 | PZ473171 |
| UnBPratySoy54 | *P. brachyurus* | Nova Ubiratã | MT | -55,2863889 | -13,2538889 | PZ487275 | PZ483885 | PZ473169 |
| UnBPratySoy55 | *P. brachyurus* and *P. zeae* | Nova Ubiratã | MT | -55,2863889 | -13,2538889 | PZ487281 | PZ483889 and PZ483798 | PZ473173 |
| UnBPratySoy56 | *P. brachyurus* | Nova Ubiratã | MT | -55,2863889 | -13,2538889 | PZ487282 | PZ483890 |  |
| UnBPratySoy57 | *P. brachyurus* and *P. zeae* | Paranatinga | MT | -54,4391667 | -13,1458333 | PZ487278 and PZ487258 | PZ483880 and PZ483778 |  |
| UnBPratySoy58 | *P. brachyurus* and *P. zeae* | Paranatinga | MT | -54,4391667 | -13,1458333 | PZ487280 | PZ483888 | PZ473172 and PZ473321 |
| UnBPratySoy59 | *P. brachyurus* | Sinop | MT | -56,005069 | -12,498722 | PZ487513 | PZ484144 | PZ473301 |
| UnBPratySoy60 | *P. brachyurus* | Querência | MT | -52,560037 | -12,234766 |  | PZ484069 |  |
| UnBPratySoy61 | *P. brachyurus* | Querência | MT | -52,560037 | -12,234766 | PZ487451 | PZ484070 | PZ473273 |
| UnBPratySoy62 | *P. brachyurus* | Querência | MT | -52,560037 | -12,234766 | PZ487454 | PZ484073 | PZ473275 |
| UnBPratySoy63 | *P. brachyurus* and *P. zeae* | Itaporã | MT | -56,6788889 | -11,4048056 | PZ487276 and PZ487260 | PZ483879 and PZ483787 | PZ473170 |
| UnBPratySoy64 | *P. brachyurus* and *P. zeae* | Itaporã | MT | -56,6788889 | -11,4048056 | PZ487279 | PZ483887 | PZ473320 |
| UnBPratySoy65 | *P. brachyurus* and *P. zeae* | Itaporã | MT | -56,6788889 | -11,4048056 | PZ487283 and PZ487268 | PZ483878 | PZ473174 |
| UnBPratySoy66 | *P. brachyurus* and *P. zeae* | Itaporã | MT | -56,6788889 | -11,4048056 | PZ487284 and PZ487259 | PZ483809 | PZ473175 |
| UnBPratySoy67 | *P. brachyurus* | União do Norte | MT | -54,419463 | -10,4308 | PZ487358 | PZ483969 |  |
| UnBPratySoy68 | *P. brachyurus* and *P. zeae* | Rio das Pedras | DF | -47,601891 | -22,8637 | PZ487307 | PZ483914 and PZ483772 |  |
| UnBPratySoy69 | *P. brachyurus* | São Sebastião | DF | -47,57909 | -16,013922 | PZ487309 | PZ483916 | PZ473196 |
| UnBPratySoy70 | *P. brachyurus* | Núcleo Rural do Rio Preto | DF | -45,9375 | -15,9922222 | PZ487362 | PZ483973 |  |
| UnBPratySoy71 | *P. brachyurus* | Barra alta | DF | -47,449802 | -15,846323 | PZ487308 | PZ483915 | PZ473195 |
| UnBPratySoy72 | *P. brachyurus* and *P. zeae* | Planaltina | DF | -47,60324 | -15,473758 | PZ487319 | PZ483926 and PZ483777 |  |
| UnBPratySoy73 | *P. brachyurus* | Planaltina | DF | -47,60324 | -15,473758 |  | PZ483932 |  |
| UnBPratySoy74 | *P. brachyurus* | Planaltina | DF | -47,60324 | -15,473758 | PZ487341 | PZ483950 |  |
| UnBPratySoy75 | *P. brachyurus* | Planaltina | DF | -46,31827 | -8,58834 | PZ487514 | PZ484145 | PZ473310 |
| UnBPratySoy76 | *P. brachyurus* and *P. zeae* | Cassilândia | GO | -51,732608 | -19,112208 | PZ487397 | PZ484009 and PZ483792 | PZ473239 |
| UnBPratySoy77 | *P. brachyurus* | Itajá | GO | -51,552416 | -19,068321 | PZ487396 | PZ484008 |  |
| UnBPratySoy78 | *P. brachyurus* and *P. zeae* | Cachoeira Dourada | GO | -49,502359 | -18,515414 | PZ487393 | PZ484006 | PZ473316 |
| UnBPratySoy79 | *P. brachyurus* | Inaciolândia | GO | -49,992403 | -18,489496 | PZ487394 | PZ484007 | PZ473238 |
| UnBPratySoy80 | *P. brachyurus* | Quirinópolis | GO | -50,450542 | -18,459499 | PZ487395 |  |  |
| UnBPratySoy81 | *P. brachyurus* | Itumbiara | GO | -49,21888 | -18,408906 | PZ487392 | PZ484005 | PZ473237 |
| UnBPratySoy82 | *P. brachyurus* | Bom Jesus | GO | -49,755929 | -18,261908 |  | PZ483894 | PZ473179 |
| UnBPratySoy83 | *P. zeae* | Bom Jesus | GO | -49,755929 | -18,261908 |  |  | PZ473327 |
| UnBPratySoy84 | *P. brachyurus* | Panamá | GO | -49,355005 | -18,182639 | PZ487391 | PZ484004 | PZ473236 |
| UnBPratySoy85 | *P. brachyurus* | Goiatuba | GO | -49,361079 | -18,01743 | PZ487390 | PZ484003 | PZ473235 |
| UnBPratySoy86 | *P. brachyurus* | Morrinhos | GO | -49,128338 | -17,754038 | PZ487388 | PZ484001 |  |
| UnBPratySoy87 | *P. brachyurus* | Morrinhos | GO | -49,128338 | -17,754038 | PZ487389 | PZ484002 | PZ473234 |
| UnBPratySoy88 | *P. brachyurus* | Ipameri | GO | -48,156687 | -17,718736 | PZ487386 | PZ483998 |  |
| UnBPratySoy89 | *P. brachyurus* | Ipameri | GO | -48,156687 | -17,718736 |  | PZ483999 |  |
| UnBPratySoy90 | *P. brachyurus* | Ipameri | GO | -48,156687 | -17,718736 | PZ487387 | PZ484000 | PZ473233 |
| UnBPratySoy91 | *P. brachyurus* | Mineiros | GO | -52,691364 | -17,479985 |  | PZ483896 | PZ473180 |
| UnBPratySoy92 | *P. brachyurus* | Mineiros | GO | -52,691364 | -17,479985 | PZ487288 | PZ483897 | PZ473181 |
| UnBPratySoy93 | *P. brachyurus* | Mineiros | GO | -52,691364 | -17,479985 | PZ487289 | PZ483898 | PZ473182 |
| UnBPratySoy94 | *P. brachyurus* and *P. zeae* | Montividiu | GO | -51,2833333 | -17,3308333 | PZ487286 | PZ483892 and PZ483811 | PZ473177 |
| UnBPratySoy95 | *P. brachyurus* and *P. zeae* | Montividiu | GO | -51,2833333 | -17,3308333 | PZ487287 | PZ483893 and PZ483810 | PZ473178 |
| UnBPratySoy96 | *P. brachyurus* and *P. zeae* | São Miguel do Passo Quatro | GO | -48,719818 | -16,979797 | PZ487292 | PZ483900 and PZ483790 | PZ473185 |
| UnBPratySoy97 | *P. brachyurus* and *P. zeae* | São Miguel do Passo Quatro | GO | -48,719818 | -16,979797 | PZ487232 | PZ483901 and PZ483775 |  |
| UnBPratySoy98 | *P. brachyurus* and *P. zeae* | São Miguel do Passo Quatro | GO | -48,719818 | -16,979797 |  | PZ483902 and PZ483776 |  |
| UnBPratySoy99 | *P. brachyurus* and *P. zeae* | Caiapônia | GO | -51,786246 | -16,82283 | PZ487290 and PZ487261 | PZ483899 and PZ483808 | PZ473183 |
| UnBPratySoy100 | *P. brachyurus* and *P. zeae* | Caiapônia | GO | -51,786246 | -16,82283 | PZ487291 and PZ487267 | PZ483895 and PZ483769 | PZ473184 |
| UnBPratySoy101 | *P. brachyurus* | Cristalina | GO | -47,606984 | -16,783951 | PZ487310 | PZ483917 | PZ473197 |
| UnBPratySoy102 | *P. brachyurus* | Cristalina | GO | -47,606984 | -16,783951 | PZ487384 | PZ483996 | PZ473232 |
| UnBPratySoy103 | *P. brachyurus* | Cristalina | GO | -47,606984 | -16,783951 | PZ487385 | PZ483997 |  |
| UnBPratySoy104 | *P. brachyurus* | Silvânia | GO | -48,601674 | -16,676893 | PZ487314 |  |  |
| UnBPratySoy105 | *P. brachyurus* | Luiziana | GO | -48,481089 | -16,3431 | PZ487302 | PZ483909 |  |
| UnBPratySoy106 | *P. brachyurus* | Luiziana | GO | -47,939189 | -16,281797 | PZ487304 | PZ483911 | PZ473193 |
| UnBPratySoy107 | *P. brachyurus* and *P. zeae* | Luiziana | GO | -47,939189 | -16,281797 | PZ487311 and PZ487252 | PZ483918 |  |
| UnBPratySoy108 | *P. brachyurus* and *P. zeae* | Luiziana | GO | -47,939189 | -16,281797 | PZ487312 and PZ487251 | PZ483919 and PZ483801 | PZ473198 |
| UnBPratySoy109 | *P. brachyurus* and *P. zeae* | Abadiânia | GO | -48,685145 | -16,218698 | PZ487293 and PZ487262 | PZ483903 and PZ483771 |  |
| UnBPratySoy110 | *P. brachyurus* | Cabeceiras | GO | -46,928225 | -15,804116 | PZ487328 | PZ483937 | PZ473208 |
| UnBPratySoy111 | *P. brachyurus* | Cabeceiras | GO | -46,928225 | -15,804116 | PZ487380 | PZ483992 |  |
| UnBPratySoy112 | *P. brachyurus* | Planaltina | GO | -47,60324 | -15,473758 | PZ487381 | PZ483993 | PZ473231 |
| UnBPratySoy113 | *P. brachyurus* | Ceres | GO | -49,534373 | -15,413348 | PZ487294 | PZ483904 | PZ473186 |
| UnBPratySoy114 | *P. brachyurus* and *P. zeae* | Ceres | GO | -49,534373 | -15,413348 | PZ487295 | PZ483905 and PZ483781 |  |
| UnBPratySoy115* | *P. brachyurus* | Ceres | GO | -49,534373 | -15,413348 | PZ487296 | PZ670768 | PZ473187 |
| UnBPratySoy116 | *P. brachyurus* | Ceres | GO | -49,534373 | -15,413348 | PZ487297 |  | PZ473188 |
| UnBPratySoy117 | *P. brachyurus* | Água Fria | GO | -47,791866 | -14,985044 | PZ487315 | PZ483921 |  |
| UnBPratySoy118 | *P. brachyurus* | Água Fria | GO | -47,791866 | -14,985044 | PZ487324 | PZ483933 | PZ473204 |
| UnBPratySoy119 | *P. brachyurus* | Barro Alto | GO | -48,910085 | -14,907931 | PZ487298 | PZ670765 | PZ473189 |
| UnBPratySoy120 | *P. brachyurus* | Barro Alto | GO | -48,910085 | -14,907931 | PZ487299 | PZ483906 | PZ473190 |
| UnBPratySoy121* | *P. brachyurus* | Barro Alto | GO | -48,910085 | -14,907931 |  |  | PZ473191 |
| UnBPratySoy122 | *P. brachyurus* | Barro Alto | GO | -48,910085 | -14,907931 | PZ487300 | PZ483907 | PZ473192 |
| UnBPratySoy123 | *P. brachyurus* | São João d`Aliança | GO | -47,522114 | -14,707152 | PZ487316 | PZ483924 | PZ473200 |
| UnBPratySoy124 | *P. brachyurus* and *P. zeae* | São João d'Aliança | GO | -47,522114 | -14,707152 | PZ487356 and PZ487256 | PZ483967 |  |
| UnBPratySoy125 | *P. brachyurus* | Alvorada do Norte | GO | -46,49246 | -14,483553 | PZ487326 | PZ483935 | PZ473206 |
| UnBPratySoy126 | *P. brachyurus* | Niquelândia | GO | -48,460186 | -14,471066 |  | PZ483923 |  |
| UnBPratySoy127 | *P. brachyurus* | Niquelândia | GO | -48,460186 | -14,471066 | PZ487322 | PZ483930 | PZ473203 |
| UnBPratySoy128 | *P. brachyurus* | Niquelândia | GO | -48,460186 | -14,471066 | PZ487323 | PZ483931 |  |
| UnBPratySoy129 | *P. brachyurus* | Porangatu | GO | -49,260625 | -13,36543 | PZ487301 | PZ483908 |  |
| UnBPratySoy130 | *P. brachyurus* | Uberaba | MG | -47,974598 | -19,769888 | PZ487436 | PZ484043 | PZ473262 |
| UnBPratySoy131 | *P. brachyurus* | Uberaba | MG | -47,974598 | -19,769888 | PZ487437 | PZ484062 |  |
| UnBPratySoy132 | *P. brachyurus* | Uberaba | MG | -47,974598 | -19,769888 | PZ487438 | PZ484044 | PZ473263 |
| UnBPratySoy133 | *P. brachyurus* | Uberaba | MG | -47,974598 | -19,769888 | PZ487439 | PZ484045 |  |
| UnBPratySoy134 | *P. brachyurus* | Prata | MG | -49,0420485 | -19,3730743 | PZ487458 | PZ484077 |  |
| UnBPratySoy135 | *P. brachyurus* | Indianópolis | MG | -47,915793 | -19,039455 |  | PZ484048 | PZ473266 |
| UnBPratySoy136 | *P. brachyurus* | Indianópolis | MG | -47,915793 | -19,039455 | PZ487442 | PZ484049 | PZ473267 |
| UnBPratySoy137 | *P. brachyurus* | Uberlândia | MG | -48,252925 | -18,946415 | PZ487440 | PZ484046 | PZ473264 |
| UnBPratySoy138 | *P. brachyurus* | Uberlândia | MG | -48,252925 | -18,946415 | PZ487441 | PZ484047 | PZ473265 |
| UnBPratySoy139 | *P. brachyurus* | Formosa de Minas | MG | -46,390197 | -18,810638 | PZ487353 | PZ483964 |  |
| UnBPratySoy140 | *P. brachyurus* | Estrela do Sul | MG | -47,692132 | -18,745392 | PZ487443 | PZ484063 | PZ473268 |
| UnBPratySoy141 | *P. brachyurus* | Abadia dos dourados | MG | -47,3872879 | -18,4852333 | PZ487444 | PZ484050 | PZ473269 |
| UnBPratySoy142 | *P. brachyurus* | Alegre | MG | -47,1983146 | -18,4208238 | PZ487445 | PZ484051 |  |
| UnBPratySoy143 | *P. brachyurus* | Alegre | MG | -47,1983146 | -18,4208238 | PZ487446 | PZ484052 | PZ473270 |
| UnBPratySoy144 | *P. brachyurus* | Claro de minas | MG | -47,0592008 | -18,1099451 | PZ487447 | PZ484064 | PZ473271 |
| UnBPratySoy145 | *P. brachyurus* | Guarda-Mor | MG | -47,0440297 | -17,6500024 | PZ487448 | PZ484066 | PZ473272 |
| UnBPratySoy146 | *P. brachyurus* | Guarda-Mor | MG | -47,0440297 | -17,6500024 | PZ487449 | PZ484067 |  |
| UnBPratySoy147 | *P. brachyurus* | Paracatu | MG | -46,975017 | -17,4607804 | PZ487450 | PZ484068 |  |
| UnBPratySoy148 | *P. brachyurus* | Unaí | MG | -46,884662 | -16,339711 | PZ487313 | PZ483920 | PZ473199 |
| UnBPratySoy149 | *P. brachyurus* and *P. zeae* | Unaí | MG | -46,884662 | -16,339711 | PZ487363 and PZ487234 | PZ483974 | PZ473222 |
| UnBPratySoy150 | *P. brachyurus* | Unaí | MG | -46,884662 | -16,339711 | PZ487364 | PZ483975 | PZ473223 |
| UnBPratySoy151 | *P. brachyurus* | Arinos | MG | -45,9375 | -15,9922222 | PZ487359 | PZ483970 |  |
| UnBPratySoy152 | *P. brachyurus* | Arinos | MG | -45,9375 | -15,9922222 | PZ487360 | PZ483971 |  |
| UnBPratySoy153 | *P. brachyurus* | Arinos | MG | -45,9375 | -15,9922222 | PZ487361 | PZ483972 | PZ473220 |
| UnBPratySoy154 | *P. brachyurus* | Buritis | MG | -46,423811 | -15,645967 | PZ487317 | PZ483925 |  |
| UnBPratySoy155 | *P. brachyurus* | Buritis | MG | -46,423811 | -15,645967 | PZ487318 |  |  |
| UnBPratySoy156 | *P. brachyurus* | Buritis | MG | -46,423811 | -15,645967 | PZ487320 | PZ483927 | PZ473201 |
| UnBPratySoy157 | *P. brachyurus* | Buritis | MG | -46,423811 | -15,645967 | PZ487325 | PZ483934 | PZ473205 |
| UnBPratySoy158 | *P. brachyurus* | Buritis | MG | -46,423811 | -15,645967 | PZ487354 | PZ483965 |  |
| UnBPratySoy159 | *P. brachyurus* | Formosa | MG | -47,284105 | -15,57905 |  | PZ483922 |  |
| UnBPratySoy160 | *P. brachyurus* and *P. zeae* | Formosa | MG | -47,284105 | -15,57905 | PZ487329 | PZ483938 | PZ473209 |
| UnBPratySoy161 | *P. brachyurus* | Serra Bonita | MG | -46,815453 | -15,211585 | PZ487305 | PZ483912 |  |
| UnBPratySoy162 | *P. brachyurus* | Serra Bonita | MG | -46,815453 | -15,211585 |  | PZ483929 |  |
| UnBPratySoy163 | *P. brachyurus* | Formoso | MG | -46,223567 | -14,984106 | PZ487455 |  |  |
| UnBPratySoy164 | *P. brachyurus* | Mundo Novo | MS | -54,28605 | -23,936829 | PZ487424 | PZ484031 | PZ473244 |
| UnBPratySoy165 | *P. brachyurus* | Itaquiraí | MS | -54,187709 | -23,475579 | PZ487420 | PZ484058 |  |
| UnBPratySoy166* | *P. brachyurus* | Itaquiraí | MS | -54,187709 | -23,475579 | PZ487421 | PZ484028 | PZ473249 |
| UnBPratySoy167 | *P. brachyurus* | Itaquiraí | MS | -54,187709 | -23,475579 | PZ487422 | PZ484029 |  |
| UnBPratySoy168 | *P. brachyurus* | Itaquiraí | MS | -54,187709 | -23,475579 | PZ487423 | PZ484030 |  |
| UnBPratySoy169 | *P. brachyurus* and *P. zeae* | Juti | MS | -54,606026 | -22,86581 | PZ487419 and PZ487235 | PZ484027 and PZ483794 | PZ473248 |
| UnBPratySoy170 | *P. brachyurus* | Caarapó | MS | -54,824913 | -22,63522 | PZ487417 | PZ484025 |  |
| UnBPratySoy171* | *P. brachyurus* | Caarapó | MS | -54,824913 | -22,63522 | PZ487418 | PZ484026 | PZ473247 |
| UnBPratySoy172 | *P. brachyurus* and *P. zeae* | Dourados | MS | -54,804816 | -22,239236 | PZ487331 and PZ487253 | PZ483940 and PZ483782 |  |
| UnBPratySoy173 | *P. brachyurus* | Dourados | MS | -54,804816 | -22,239236 |  | PZ484057 |  |
| UnBPratySoy174 | *P. brachyurus* | Dourados | MS | -54,804816 | -22,239236 | PZ487416 | PZ484024 |  |
| UnBPratySoy175 | *P. brachyurus* | Douradina | MS | -54,5437021 | -22,0120247 | PZ487415 | PZ484023 | PZ473246 |
| UnBPratySoy176 | *P. brachyurus* | Rio Brilhante | MS | -54,525303 | -21,78606 | PZ487413 | PZ484021 |  |
| UnBPratySoy177 | *P. brachyurus* | Rio Brilhante | MS | -54,525303 | -21,78606 | PZ487414 | PZ484022 | PZ473245 |
| UnBPratySoy178 | *P. brachyurus* | Maracaju | MS | -55,187042 | -21,632598 | PZ487330 | PZ483939 |  |
| UnBPratySoy179 | *P. brachyurus* | Sidrolândia | MS | -54,963504 | -20,939487 | PZ487411 | PZ484019 |  |
| UnBPratySoy180 | *P. brachyurus* | Sidrolândia | MS | -54,963504 | -20,939487 | PZ487412 | PZ484020 |  |
| UnBPratySoy181 | *P. brachyurus* | Campo Grande | MS | -54,566194 | -20,535709 | PZ487409 |  |  |
| UnBPratySoy182 | *P. brachyurus* | Campo Grande | MS | -54,566194 | -20,535709 | PZ487410 | PZ484018 |  |
| UnBPratySoy183 | *P. brachyurus* | Bandeirantes | MS | -54,335889 | -19,887869 |  | PZ484065 |  |
| UnBPratySoy184 | *P. brachyurus* | Bandeirantes | MS | -54,335889 | -19,887869 | PZ487408 | PZ484054 | PZ473243 |
| UnBPratySoy185 | *P. brachyurus* and *P. zeae* | Bandeirantes | MS | -54,335889 | -19,887869 |  | PZ484055 and PZ483795 |  |
| UnBPratySoy186 | *P. brachyurus* | Camapuã | MS | -54,035803 | -19,543378 | PZ487407 | PZ484017 | PZ473242 |
| UnBPratySoy187 | *P. brachyurus* | Paraíso das Águas | MS | -53,019912 | -19,03222 | PZ487404 | PZ484015 | PZ473241 |
| UnBPratySoy188* | *P. brachyurus* | Paraíso das Águas | MS | -53,019912 | -19,03222 |  | PZ484016 |  |
| UnBPratySoy189 | *P. brachyurus* | Paraíso das Águas | MS | -53,019912 | -19,03222 | PZ487405 | PZ484056 |  |
| UnBPratySoy190 | *P. brachyurus* and *P. zeae* | Chapadão do Sul | MS | -52,622561 | -18,797896 | PZ487398 | PZ484010 | PZ473240 and PZ473323 |
| UnBPratySoy191 | *P. brachyurus* and *P. zeae* | Costa Rica | MS | -53,12627 | -18,58135 | PZ487399 | PZ484011 | PZ473319 |
| UnBPratySoy192 | *P. brachyurus* | Costa Rica | MS | -53,12627 | -18,58135 | PZ487400 | PZ484012 |  |
| UnBPratySoy193 | *P. brachyurus* | Costa Rica | MS | -53,12627 | -18,58135 | PZ487401 | PZ484053 |  |
| UnBPratySoy194 | *P. brachyurus* and *P. zeae* | Costa Rica | MS | -53,12627 | -18,58135 | PZ487406 and PZ487249 | PZ484013 |  |
| UnBPratySoy195 | *P. brachyurus* | Costa Rica | MS | -53,12627 | -18,58135 | PZ487403 | PZ484014 |  |
| UnBPratySoy196 | *P. brachyurus* and *P. zeae* | São Mateus | ES | -40,069266 | -18,66628 | PZ487254 | PZ483959 and PZ483773 |  |
| UnBPratySoy197 | *P. brachyurus* and *P. zeae* | Pinheiro | ES | -40,228843 | -18,425358 | PZ487349 and PZ487248 | PZ483960 and PZ483780 | PZ473324 |
| UnBPratySoy198 | *P. brachyurus* | Pinheiro | ES | -40,228843 | -18,425358 | PZ487350 | PZ483961 |  |
| UnBPratySoy199 | *P. brachyurus* and *P. zeae* | Montanha | ES | -40,366659 | -18,104093 | PZ487351 and PZ487250 | PZ483962 | PZ473218 |
| UnBPratySoy200 | *P. brachyurus* and *P. zeae* | Montanha | ES | -40,366659 | -18,104093 | PZ487352 and PZ487236 | PZ483963 | PZ473219 |
| UnBPratySoy201 | *P. brachyurus* | Itararé | SP | -49,3031016 | -24,1004866 |  | PZ484110 |  |
| UnBPratySoy202 | *P. brachyurus* | Itaberá | SP | -49,2797557 | -23,6281353 | PZ487488 | PZ484111 | PZ473291 |
| UnBPratySoy203 | *P. brachyurus* | Avaré | SP | -48,8805008 | -23,0406556 |  | PZ484112 |  |
| UnBPratySoy204 | *P. brachyurus* | Santa Cruz do Rio Pardo | SP | -49,565997 | -22,841451 | PZ487432 | PZ484037 |  |
| UnBPratySoy205 | *P. brachyurus* | Santa Cruz do Rio Pardo | SP | -49,5855556 | -22,8202778 | PZ487382 | PZ483994 |  |
| UnBPratySoy206 | *P. brachyurus* | Santa Cruz do Rio Pardo | SP | -49,5855556 | -22,8202778 | PZ487383 | PZ483995 |  |
| UnBPratySoy207 | *P. brachyurus* | Nantes | SP | -51,2609763 | -22,6178918 | PZ487467 | PZ484086 |  |
| UnBPratySoy208 | *P. brachyurus* and *P. zeae* | Piracicaba | SP | -47,637284 | -22,606585 | PZ487347 | PZ483957 and PZ483774 | PZ473216 |
| UnBPratySoy209 | *P. brachyurus* | Iepê | SP | -51,0550344 | -22,5629968 |  | PZ484085 |  |
| UnBPratySoy210* | *P. brachyurus* | Rancharia | SP | -50,8963186 | -22,1869447 | PZ487465 | PZ484083 | PZ473279 |
| UnBPratySoy211 | *P. brachyurus* | Rancharia | SP | -50,8963186 | -22,1869447 | PZ487466 | PZ484084 | PZ473280 |
| UnBPratySoy212 | *P. brachyurus* | Araraquara | SP | -48,168387 | -21,808455 | PZ487433 | PZ484038 |  |
| UnBPratySoy213* | *P. brachyurus* | Clementina | SP | -50,4398272 | -21,537001 | PZ487464 | PZ484094 |  |
| UnBPratySoy214 | *P. brachyurus* | Cravinhos | SP | -47,732521 | -21,338727 | PZ487434 | PZ484039 | PZ473259 |
| UnBPratySoy215 | *P. brachyurus* | Araçatuba | SP | -50,4588767 | -21,10632 | PZ487462 | PZ484081 |  |
| UnBPratySoy216 | *P. brachyurus* | Araçatuba | SP | -50,4588767 | -21,10632 | PZ487463 | PZ484082 | PZ473278 |
| UnBPratySoy217 | *P. brachyurus* and *P. zeae* | Santo Antônio do Aracanguá | SP | -50,4573289 | -21,0233983 | PZ487461 | PZ484080 and PZ483783 |  |
| UnBPratySoy218 | *P. brachyurus* | Nova Aliança | SP | -49,498695 | -21,010394 | PZ487303 | PZ483910 |  |
| UnBPratySoy219 | *P. brachyurus* and *P. zeae* | Bebedouro | SP | -48,459201 | -20,9112505 | PZ487244 | PZ484113 and PZ483802 | PZ473326 |
| UnBPratySoy220 | *P. brachyurus* and *P. zeae* | Orlândia | SP | -47,889322 | -20,717557 |  | PZ484040 and PZ483807 |  |
| UnBPratySoy221 | *P. brachyurus* | Pinheiros | SP | -47,8384062 | -20,5028122 |  | PZ484041 | PZ473260 |
| UnBPratySoy222 | *P. brachyurus* | Jales | SP | -50,5124281 | -20,3785686 | PZ487460 | PZ484079 |  |
| UnBPratySoy223 | *P. brachyurus* | Guaíra | SP | -48,261509 | -20,3125736 | PZ487489 |  |  |
| UnBPratySoy224 | *P. brachyurus* and *P. zeae* | Miguelópolis | SP | -48,1892632 | -20,1097925 | PZ487238 | PZ484114 and PZ483785 |  |
| UnBPratySoy225 | *P. brachyurus* | Aramina | SP | -47,789457 | -20,086318 | PZ487435 | PZ484042 | PZ473261 |
| UnBPratySoy226* | *P. brachyurus* | Ouroeste | SP | -50,4013717 | -19,8974175 | PZ487459 | PZ484078 | PZ473277 |
| UnBPratySoy227 | *P. brachyurus* | Garapuava | PR | -51,417736 | -25,438311 | PZ487306 | PZ483913 | PZ473194 |
| UnBPratySoy228 | *P. brachyurus* | Candói | PR | -52,0120408 | -25,426204 | PZ487484 | PZ484104 |  |
| UnBPratySoy229 | *P. brachyurus* | Candói | PR | -52,0120408 | -25,426204 | PZ487485 | PZ484105 |  |
| UnBPratySoy230 | *P. brachyurus* | Virmond | PR | -52,2782357 | -25,3730513 | PZ487483 | PZ484103 | PZ473292 |
| UnBPratySoy231 | *P. brachyurus* | Guarapuava | PR | -51,50007 | -25,372774 | PZ487511 | PZ484142 | PZ473304 |
| UnBPratySoy232 | *P. brachyurus* | Laranjeiras do Sul | PR | -52,4397624 | -25,3721916 | PZ487482 | PZ484102 | PZ473290 |
| UnBPratySoy233 | *P. brachyurus* | Nova Laranjeiras | PR | -52,6535565 | -25,2202908 | PZ487480 | PZ484100 |  |
| UnBPratySoy234 | *P. brachyurus* | Nova Laranjeiras | PR | -52,6535565 | -25,2202908 | PZ487481 | PZ484101 | PZ473289 |
| UnBPratySoy235 | *P. brachyurus* | Ponta Grossa | PR | -50,3678912 | -25,0788067 |  | PZ484106 |  |
| UnBPratySoy236 | *P. brachyurus* | Carambeí | PR | -50,1020409 | -24,9320113 | PZ487486 | PZ484107 |  |
| UnBPratySoy237 | *P. brachyurus* | Cascavel | PR | -53,3455456 | -24,8819605 | PZ487479 | PZ484099 |  |
| UnBPratySoy238 | *P. brachyurus* | Corbélia | PR | -53,2959054 | -24,77009 | PZ487478 | PZ484098 | PZ473288 |
| UnBPratySoy239* | *P. brachyurus* | Ouro Verde do Piquiri | PR | -53,2081253 | -24,6329716 | PZ487477 | PZ484097 | PZ473287 |
| UnBPratySoy240 | *P. brachyurus* and *P. zeae* | Castro | PR | -49,9591448 | -24,6151536 |  | PZ484108 and PZ670763 | PZ473293 |
| UnBPratySoy241 | *P. brachyurus* | Nova Aurora | PR | -53,2286141 | -24,4585538 | PZ487476 | PZ484093 |  |
| UnBPratySoy242 | *P. brachyurus* | Quarto Centenário | PR | -53,1250306 | -24,3360777 | PZ487475 | PZ484096 |  |
| UnBPratySoy243 | *P. brachyurus* | Jaguariaíva | PR | -49,6376656 | -24,2066917 | PZ487487 | PZ484109 | PZ473314 |
| UnBPratySoy244 | *P. zeae* | Cruzeiro do Oeste | PR | -52,9879618 | -23,7629605 | PZ671226 | PZ483797 |  |
| UnBPratySoy245 | *P. brachyurus* | Cruzeiro do Oeste | PR | -52,9879618 | -23,7629605 | PZ487474 | PZ484092 | PZ473286 |
| UnBPratySoy246 | *P. brachyurus* and *P. zeae* | Jussara | PR | -52,4057107 | -23,6024239 | PZ487473 and PZ487240 | PZ484091 |  |
| UnBPratySoy247 | *P. brachyurus* | Apucarana | PR | -51,441989 | -23,560289 | PZ487426 | PZ484059 | PZ473252 |
| UnBPratySoy248 | *P. brachyurus* | Marialva | PR | -51,819123 | -23,476778 | PZ487425 | PZ484032 | PZ473250 |
| UnBPratySoy249* | *P. brachyurus* | Marialva | PR | -51,819123 | -23,476778 |  |  | PZ473251 |
| UnBPratySoy250 | *P. brachyurus* | Rolândia | PR | -51,377523 | -23,315498 | PZ487427 |  | PZ473253 |
| UnBPratySoy251 | *P. brachyurus* | Ibiporã | PR | -51,058165 | -23,275761 | PZ487428 | PZ484033 |  |
| UnBPratySoy252 | *P. brachyurus* | Jataizinho | PR | -50,973907 | -23,259258 | PZ487429 | PZ484060 | PZ473254 |
| UnBPratySoy253 | *P. brachyurus* | Uraí | PR | -50,79032 | -23,211259 |  |  | PZ473257 |
| UnBPratySoy254 | *P. brachyurus* | Cornélio Procópio | PR | -50,639669 | -23,184697 | PZ487430 | PZ484034 | PZ473256 |
| UnBPratySoy255 | *P. brachyurus* | Santa Mariana | PR | -50,505321 | -23,152426 | PZ487431 | PZ484035 | PZ473255 |
| UnBPraty256 | *P. brachyurus* | Barra do Jacaré | PR | -50,181705 | -23,116008 |  | PZ484036 |  |
| UnBPratySoy257 | *P. brachyurus* | Barra do Jacaré | PR | -50,181705 | -23,116008 |  | PZ484061 | PZ473258 |
| UnBPratySoy258 | *P. brachyurus* | Guaraci | PR | -51,6661876 | -22,9701652 | PZ487471 | PZ484095 | PZ473284 |
| UnBPratySoy259 | *P. brachyurus* | Guaraci | PR | -51,6661876 | -22,9701652 | PZ487472 | PZ484090 | PZ473285 |
| UnBPratySoy260 | *P. brachyurus* | Centenario do Sul | PR | -51,5135828 | -22,8090276 | PZ487469 | PZ484088 | PZ473282 |
| UnBPratySoy261 | *P. brachyurus* | Centenario do Sul | PR | -51,5135828 | -22,8090276 | PZ487470 | PZ484089 | PZ473283 |
| UnBPratySoy262 | *P. brachyurus* | Porecatu | PR | -51,3697586 | -22,6957653 | PZ487468 | PZ484087 | PZ473281 |
| UnBPratySoy263 | *P. brachyurus* and *P. zeae* | Campos Novos | SC | -51,222682 | -27,398826 | PZ487504 | PZ484135 | PZ473309 and PZ473335 |
| UnBPratySoy264 | *P. brachyurus* and *P. zeae* | Curitibanos | SC | -50,577186 | -27,295054 | PZ487505 | PZ484136 and PZ483805 | PZ473336 |
| UnBPratySoy265 | *P. brachyurus* and *P. zeae* | Chapecó | SC | -52,647864 | -27,099547 | PZ487501 | PZ484132 and PZ483788 |  |
| UnBPratySoy266 | *P. brachyurus* and *P. zeae* | Xanxerê | SC | -52,412423 | -26,876178 | PZ487502 and PZ487242 | PZ484133 | PZ473299 and PZ473332 |
| UnBPratySoy267 | *P. brachyurus* and *P. zeae* | Caçador | SC | -51,007862 | -26,78161 | PZ487506 | PZ484137 | PZ473303 and PZ473339 |
| UnBPratySoy268 | *P. brachyurus* and *P. zeae* | São Miguel do Oeste | SC | -53,519651 | -26,738787 | PZ487503 | PZ484134 | PZ473302 and PZ473333 |
| UnBPratySoy269 | *P. brachyurus* | Canoinhas | SC | -50,397359 | -26,180815 |  | PZ484138 |  |
| UnBPratySoy270 | *P. brachyurus* and *P. zeae* | Mafra | SC | -49,818836 | -26,13028 | PZ487507 | PZ484139 | PZ473305 and PZ473337 |
| UnBPratySoy271 | *P. brachyurus* | Dom pedrito | RS | -54,659757 | -30,975726 | PZ487499 | PZ484128 | PZ473300 |
| UnBPratySoy272 | *P. brachyurus*, *P. zeae*, and *P. penetrans* | Sant'Ana do Livramento | RS | -55,475856 | -30,860829 | PZ487494 and PZ487231 | PZ484124 and PZ483756 | PZ473313 and PZ473329 |
| UnBPratySoy273 | *P. brachyurus* | São Gabriel | RS | -54,320429 | -30,34705 | PZ487498 | PZ484127 |  |
| UnBPratySoy274 | *P. brachyurus* | Cachoeira do Sul | RS | -52,953948 | -30,197751 | PZ487496 | PZ484118 | PZ473298 |
| UnBPratySoy275 | *P. brachyurus* and *P. zeae* | Santa Maria | RS | -53,69861 | -29,708945 | PZ487241 | PZ484120 and PZ483804 | PZ473334 |
| UnBPratySoy276 | *P. brachyurus* | Manoel Viana | RS | -55,474714 | -29,589584 | PZ487495 | PZ484125 |  |
| UnBPratySoy277 | *P. brachyurus* and *P. zeae* | Julio de Castilho | RS | -53,683564 | -29,236254 | PZ487490 and PZ487239 | PZ484115 | PZ473296 and PZ473328 |
| UnBPratySoy278 | *P. brachyurus* and *P. zeae* | Tupanciretã | RS | -53,846672 | -29,091553 | PZ487491 | PZ484116 and PZ483786 |  |
| UnBPratySoy279 | *P. brachyurus* and *P. zeae* | Monte Alegre dos Campos | RS | -50,808346 | -28,705232 |  | PZ484130 and PZ483803 | PZ473330 |
| UnBPratySoy280 | *P. brachyurus* | Cruz Alta | RS | -53,614166 | -28,656347 |  | PZ484117 |  |
| UnBPratySoy281 | *P. brachyurus* | Joia | RS | -54,128475 | -28,648945 | PZ487492 | PZ484122 | PZ473312 |
| UnBPratySoy282 | *P. brachyurus* and *P. zeae* | Boa vista do Cadeado | RS | -53,815201 | -28,584418 | PZ487500 and PZ671227 | PZ484121 and PZ483806 | PZ473294 |
| UnBPratySoy283 | *P. brachyurus* | Santa Bárbara do Sul | RS | -53,232486 | -28,387745 | PZ487357 | PZ483968 |  |
| UnBPratySoy284 | *P. brachyurus* | Santa Bárbara do Sul | RS | -53,302313 | -28,383757 |  | PZ484119 |  |
| UnBPratySoy285 | *P. brachyurus* | Muitos Capões | RS | -51,1824 | -28,320218 | PZ487332 | PZ483941 |  |
| UnBPratySoy286 | *P. brachyurus* | Passo Fundo | RS | -52,412005 | -28,294004 | PZ487493 | PZ484123 | PZ473295 |
| UnBPratySoy287 | *P. brachyurus* | Santo Ângelo | RS | -54,30522 | -28,275382 | PZ487343 | PZ483953 |  |
| UnBPratySoy288* | *P. brachyurus* | Santo Ângelo | RS | -54,30522 | -28,275382 | PZ487346 | PZ483956 |  |
| UnBPratySoy289 | *P. brachyurus* and *P. zeae* | Santo Ângelo | RS | -54,30522 | -28,275382 |  | PZ484131 and PZ483784 | PZ473168 and PZ473331 |
| UnBPratySoy290 | *P. brachyurus* | Santo Ângelo | RS | -54,30522 | -28,275382 | PZ487510 |  |  |
| UnBPratySoy291 | *P. brachyurus* and *P. zeae* | Lagoa Vermelha | RS | -51,525634 | -28,212427 | PZ487508 and PZ487233 | PZ484140 | PZ473307 and PZ473338 |
| UnBPratySoy292 | *P. brachyurus* | Giruá | RS | -54,330663 | -28,034693 | PZ487345 | PZ483955 | PZ473215 |
| UnBPratySoy293 | *P. brachyurus* | Palmeira das Missões | RS | -53,299461 | -27,914454 | PZ487497 | PZ484126 | PZ473311 |
| UnBPratySoy294* | *P. brachyurus* | Santa Rosa | RS | -54,461838 | -27,871303 | PZ487509 | PZ484141 | PZ473297 |
| UnBPratySoy295* | *P. brachyurus* | Santo Augusto | RS | -53,765923 | -27,854002 | PZ487344 | PZ483954 |  |
| UnBPratySoy296 | *P. brachyurus* and *P. zeae* | Erechim | RS | -52,272562 | -27,648323 | PZ487243 | PZ484129 |  |
|  |  |  |  |  |  |  |  |  |
